# Superspace description of wagnerite-group minerals (Mg,Fe,Mn)_2_(PO_4_)(F,OH)

**DOI:** 10.1107/S2052520613031247

**Published:** 2014-03-04

**Authors:** Biljana Lazic, Thomas Armbruster, Christian Chopin, Edward S. Grew, Alain Baronnet, Lukas Palatinus

**Affiliations:** a Mineralogical Crystallography, Institute of Geological Sciences, University of Bern, Freie­strasse 3, 3012 Bern, Switzerland; b Laboratoire de Géologie, Ecole Normale Supérieure – CNRS, 24 Rue Lhomond, 75231 Paris, France; c School of Earth and Climate Sciences, University of Maine, Orono, Maine 04469-5790, United States; d Aix-Marseille Université, CNRS, CINaM, UMR 7325, 13288 Marseille, France; e Institute of Physics of the Academy of Sciences of the Czech Republic, Na Slovance 2, 18221 Prague, Czech Republic

**Keywords:** wagnerite, modulated structure, superspace, unified model, triplite

## Abstract

Reinvestigation of more than 40 samples of minerals belonging to the wagnerite group (Mg, Fe, Mn)_2_(PO_4_)(F,OH) from diverse geological environments worldwide, using single-crystal X-ray diffraction analysis, showed that most crystals have incommensurate structures and, as such, are not adequately described with known polytype models (2*b*), (3*b*), (5*b*), (7*b*) and (9*b*). Therefore, we present here a unified superspace model for the structural description of periodically and aperiodically modulated wagnerite with the (3+1)-dimensional superspace group *C*2/*c*(0β0)*s*0 based on the average triplite structure with cell parameters *a* ≃ 12.8, *b* ≃ 6.4, *c* ≃ 9.6 Å, β ≃ 117° and the modulation vectors **q** = *β* b*. The superspace approach provides a way of simple modelling of the positional and occupational modulation of Mg/Fe and F/OH in wagnerite. This allows direct comparison of crystal properties.

## Abstract 2

A unified superspace model, based on average triplite structure, for the description of different modulation periodicities of wagnerite and related phases

## 1. Introduction   

**Figure 1:**
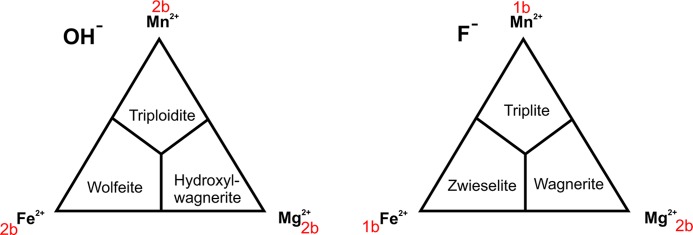
Compositional diagrams showing the two groups of phosphate minerals with the formula *M*_2_(PO_4_)*X*, where *M* = Mg^2+^, Fe^2+^, Mn^2+^ and *X* ^−^ = F, OH. Red lettering indicates structure type.

Wagnerite, first described by Fuchs (1821 ▶), is a relatively rare accessory mineral in metamorphic rocks, but occurrences in granite pegmatites and the Zechstein salt deposits have also been reported (Anthony *et al.*, 2000 ▶). Depending on chemical composition, crystals can be translucent to nearly opaque, with a wide variety of colours: colourless, white, yellowish, orange, flesh red, pink and green (Palache *et al.*, 1951 ▶, and references therein). Ideally Mg_2_(PO_4_)F, wagnerite, is better described with the general formula Mg_2 − *x*_(Fe, Mn, Ca, Ti…)_*x*_(PO_4_)(F,OH,O) because of an extensive solid solution with related minerals containing Fe^2+^, Mn^2+^ and OH (Fig. 1 ▶). Pitra *et al.* (2008 ▶) reported distinct chemical zoning in wagnerite grains: a decrease of Fe [from 0.16 to 0.08 per formula unit (p.f.u.)] and an associated increase of F (0.46–1 p.f.u.), from the centre toward the rims of the grains. When Fe^3+^ substitutes Mg^2+^, charge balance requires more negative charge at the anion site, and thus O substitutes for F and OH, as in stanekite (Fe^3+^, Mn^2+^, Fe^2+^, Mg)_2_(PO_4_)O (Keller *et al.*, 2006 ▶).

The structure of wagnerite was first solved by Coda *et al.* (1967 ▶) from single-crystal X-ray data [*P*2_1_/*c*, *a* = 9.44 (7), *b* = 12.679 (8), *c* = 11.957 (9) Å, β = 108.18 (9)°]. Another four wagnerite structure types, with different *b* periodicity (*b* ≃ 19, *b* ≃ 32, *b* ≃ 45 and *b* ≃ 57 Å) have been reported (Coda *et al.*, 1967 ▶; Ren *et al.*, 2003 ▶; Chopin, Armbruster & Leyx, 2003 ▶; Armbruster *et al.*, 2008 ▶). The close structural relationship between various stacking variants of wagnerite and *e.g.* triplite (Mn,Fe)_2_(PO_4_)F (Waldrop, 1969 ▶) with *b* = 6.45 Å led to the proposal of naming wagnerite as a polytypic series based on the triplite cell. Thus, wagnerite with 2*b* ≃ 13 Å was named wagnerite-*Ma2b*, and *e.g.* with 9*b* ≃ 57 Å wagnerite-*Ma9bc* (Burke & Ferraris, 2004 ▶).

Our structural reinvestigation of different wagnerite samples showed that the assumed *b* periodicity often displays small but significant deviations from commensurate values. Moreover, refinement of the few commensurately modulated wagnerite structures, especially with a 7*b* (*b* = 45 Å) or 9*b* (*b* = 57 Å) supercell, with occupational and positional modulation of Mg/Fe/Mn and F/OH, is much more efficient using a superspace approach. Thus, the aim of this paper is to present a unique superspace model for the structural description of both commensurately and incommensurately modulated wagnerites.

### 1.1. Origin of modulation in wagnerite   

The partial replacement of Mg^2+^ (0.72 Å) by Fe^2+^ (0.78 Å), Mn^2+^ (0.83 Å), Ca^2+^ (1.00 Å), Ti^4+^ (0.61 Å) or Fe^3+^ (0.65 Å) (Shannon & Prewitt, 1969 ▶) in the structure of wagnerite, as well as partial F ↔ OH substitution, causes significant variations of bond lengths. As a consequence, individual coordination polyhedra around cation sites are locally modified regarding coordination number and geometry and this may affect the geometry of the whole structure. The key to understanding the influence of chemical composition on structural periodicity in wagnerite is its structural relation to other minerals such as triplite (Mn, Fe)_2_(PO_4_)F (Waldrop, 1969 ▶) and triploidite (Mn, Fe)_2_(PO_4_)OH (Waldrop, 1968 ▶).

Based on chemical compositions and crystal morphologies, Brush & Dana (1878 ▶) suggested that the OH group in triploidite plays a corresponding role as fluorine in wagnerite and triplite. The single-crystal X-ray data obtained for wagnerite by Coda *et al.* (1967 ▶) and for triploidite by Waldrop (1968 ▶) have revealed the same features: reflections on procession photographs could be divided by intensity into two groups. If only strong reflections are indexed, then the resulting unit cell corresponds to that of triplite (*a* ≃ 12.05, *b* ≃ 6.45, *c* ≃ 9.9 Å, β = 105–107 °) with *I*2/*c* symmetry. Indexing of all reflections leads to a cell of lower symmetry (*P*2_1_/*c*) with doubled *b* parameter (*b* ≃ 13 Å) compared with triplite.

Pending a formal classification, we suggest that structurally related minerals having the general formula *M*_2_(PO_4_)F and *M*_2_(PO_4_)OH could be placed into two groups within a triplite supergroup (Fig. 1 ▶). Members of the OH-dominant group belong to the (2*b*) structure type, whereas in the F-dominant group only wagnerite has the (2*b*) structure type with triplite Mn_2_(PO_4_)F and zwieselite Fe_2_(PO_4_)F belonging to the (1*b*) structure type. These minerals form an extensive solid-solution series with each other. Table 1 ▶ summarizes the unit-cell dimensions of synthetic and natural end-members with different *b* periodicities. To be consistent with our model for wagnerite, unit-cell parameters are given in a different setting than originally reported. Transformation matrices are given in a footnote to Table 1 ▶. The (1*b*) structure type with space group *C*2*/c* is observed in the synthetic end-members Mn_2_(PO_4_)F (Rea & Kostiner, 1972 ▶) and Fe_2_(PO_4_)F (Yakubovich *et al.*, 1978 ▶) and F-dominant triplite and zwieselite samples (Armbruster *et al.*, 2008 ▶) such as Mn_0.95_Fe_0.25_Mg_0.7_PO_4_F (Waldrop, 1969 ▶) or 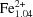
Mn_0.86_(Fe^3+^, Ca, Mg, Ti^4+^, Zn)_0.1_PO_4_F_0.85_OH_0.15_ (Origlieri, 2005 ▶).

**Figure 2:**
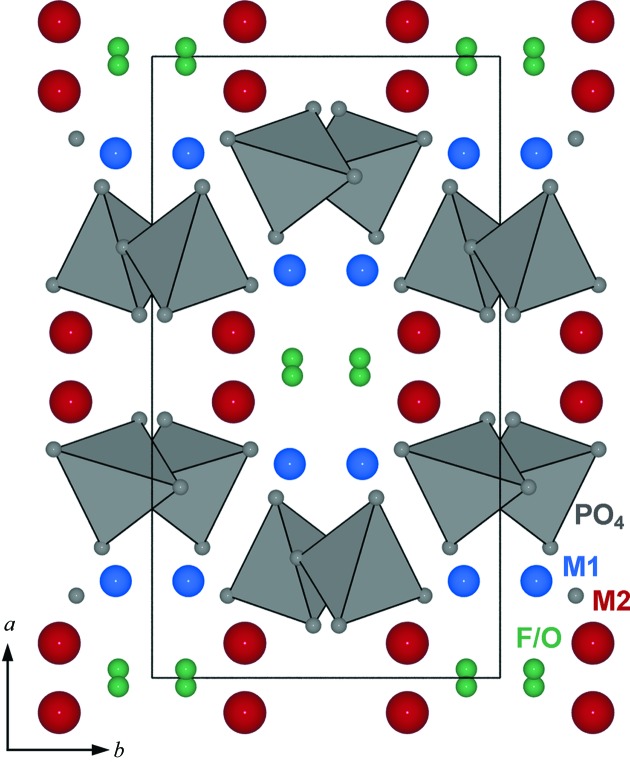
The (1*b*) structure type observed in *M*_2_(PO_4_)*X* minerals (*C*2/*c*), where *M* = Fe^2+^, Mn^2+^ and *X* **^−^** = F (Rea & Kostiner, 1972 ▶; Yakubovich *et al.*, 1978 ▶). PO_4_ units are displayed as grey tetrahedra, five- or six-coordinated cations as red spheres and F/O(H) atoms as green spheres.

The (1*b*) structure has two symmetrically independent *M*-cation positions forming *M*O_4_F_2_ polyhedra and one PO_4_ tetrahedron (Fig. 2 ▶). Fluorine occupies a compromise position and has distorted tetrahedral coordination by four *M* cations. In this context a ‘compromise position’ means that F occupies a site enabling sixfold coordination of *M*1 and *M*2, but one *M*—F bond in each octahedron is strongly elongated.

The structure of the (2*b*) type with the *P*2_1_/*n* space group is represented by three end-members: Mg_2_(PO_4_)F (this paper), Mg_2_(PO_4_)OH (Raade & Rømming, 1986 ▶) and Fe_2_(PO_4_)OH (Hatert, 2007 ▶) and minerals with intermediate composition, such as wagnerite (Mg, Fe)_2_(PO_4_)F (Coda *et al.*, 1967 ▶), hydroxylwagnerite (Mg, Fe)_2_(PO_4_)OH (Brunet *et al.*, 1998 ▶; Chopin *et al.*, 2004 ▶), triploidite Mn_1.5_Fe_0.5_(PO_4_)OH (Waldrop, 1968 ▶) and Mg-rich wolfeite (Fe, Mg)_2_(PO_4_)OH (Kolitsch, 2003 ▶). The unit-cell parameters of Mn_1.5_Fe_0.5_(PO_4_)OH (Waldrop, 1968 ▶) are also listed in Table 1 ▶, because pure Mn_2_(PO_4_)OH has not been reported so far.

**Figure 3:**
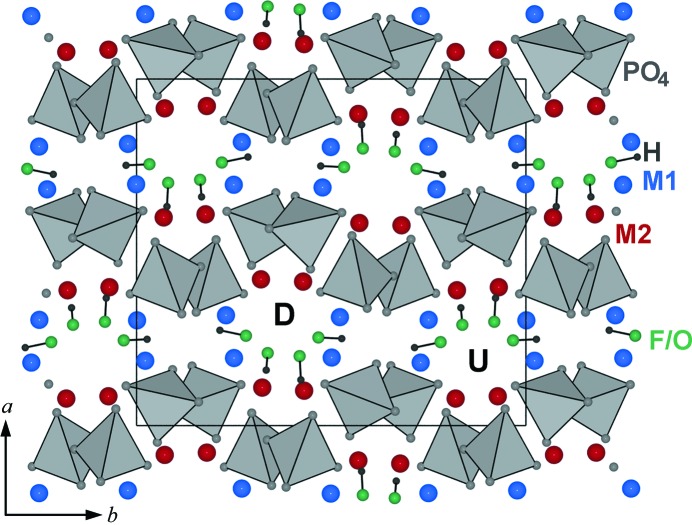
The (2*b*) structure type, observed in *M*_2_(PO_4_)*X* minerals, where *M* = Mg^2+^ and *X* ^−^ = OH, F or *M* = Fe^2+^, Mn^2+^ and *X* ^−^ = OH. Two distinct arc-like configurations of F/O atoms are labelled up (U) and down (D). The example represents synthetic hydroxylwagnerite Mg_2_(PO_4_)OH (Raade & Rømming, 1986 ▶); hydrogen bonds (donor green, hydrogen black spheres) are shown as solid lines.

Due to doubling of the *b* axis and a decrease in multiplicity of the general positions from 8 in *C*2*/c* [(1*b*) type] to 4 in *P*2_1_/*n* [(2*b*) type], the (2*b*) structure displays four times more symmetry-independent sites than (1*b*). Thus there are eight cation sites (*M*) and four F sites. Nevertheless, the (2*b*) structure type preserves the same arrangement of cations and O atoms as (1*b*), but differs in the arrangement of F atoms (Fig. 3 ▶). In contrast to the (1*b*) structure, F atoms are moved out of the compromise position and appear in the *ab* plane as two distinct arc-like configurations labelled up (U) and down (D). This arc-like arrangement is only an optical illusion originating from the special projection. Actually F sites are not coplanar. As a consequence of the shift, F atoms in (2*b*) structures are in threefold coordination. Furthermore, half of the *M* sites are five-coordinated (*M*O_4_F) and the other half are six-coordinated (*M*O_4_F_2_). Interestingly, wagnerite and hydroxylwagnerite have the same symmetry (*P*2_1_/*n*), whereas the Fe^2+^ and Mn^2+^ fluorine and hydroxyl end-members are distinct in symmetry (*C*2/*c* and *P*2_1_/*n*, respectively). The influence of the F ↔ OH substitution on unit-cell dimensions can be recognized by comparing end-members Mg_2_(PO_4_)F (this paper) with Mg_2_(PO_4_)OH (Raade & Rømming, 1986 ▶). The four fluorine positions in Mg_2_(PO_4_)F are replaced by four OH groups, thus the geometry of *M*1 and *M*2 polyhedra is preserved. In addition to the three bonds to Mg [equivalent to Mg—F in Mg_2_(PO_4_)F], O acts as a hydrogen-bond donor. The position of hydrogen is fixed by a weak hydrogen bond to an O acceptor (within 2.1 Å). Two of four such O—H bonds (0.95 Å) are oriented opposite each other, approximately parallel to *b* (Fig. 3 ▶), resulting in an increase of *b* from 12.755 Å in pure Mg_2_(PO_4_)F to 12.859 Å in pure Mg_2_(PO_4_)OH. Two other O—H bonds are oriented diagonally between *a* and *c*, causing only a slight increase of cell parameters.

The influence of the size of *M* ^2+^ cations, *e.g.* in Mg_2_(PO_4_)F (2*b*) *versus* Mn_2_(PO_4_)F (1*b*) and OH or F anions, *e.g.* in Fe_2_(PO_4_)F (1*b*) *versus* Fe_2_(PO_4_)OH (2*b*), on the structural periodicity or modulation is evident, especially for end-members. In the case of F end-members, large *M* ^2+^ radii seem to stabilize the (1*b*) structure, also confirmed by the structure of Cd_2_(PO_4_)F (Rea & Kostiner, 1974 ▶) with an octahedral Cd^2+^ radius of 0.95 Å (Shannon, 1976 ▶), whereas cations with a small octahedral radius (Mg 0.72 Å, Zn 0.74 Å) stabilize the (2*b*) structure characteristic of wagnerite and synthetic Zn_2_(PO_4_)F (Taasti *et al.*, 2002 ▶). An exception is represented by Cu_2_(PO_4_)F (Rea & Kostiner, 1976 ▶). As a result of the Jahn–Teller effect (Jahn & Teller, 1937 ▶) for Cu^2+^, Cu_2_PO_4_F (Rea & Kostiner, 1976 ▶) has (1*b*) triplite-like structure, although the ionic radius of Cu^2+^ is 0.73 Å, similar to Mg with 0.72 Å. Cu_2_(PO_4_)OH, with a structure corresponding to the triplite supergroup, has not been reported so far.

Until 2008, among 38 investigated wagnerite samples and related minerals (*e.g.* triplite), six structural polytypes have been refined from single-crystal data and imaged by high-resolution transmission electron microscopy (HRTEM; Armbruster *et al.*, 2008 ▶). The (1*b*) structure type was confirmed only for triplite–zwieselite samples. The remaining five polytypes (2*b*), (3*b*), (5*b*), (7*b*) and (9*b*) were identified in compositionally complex wagnerite.

### 1.2. Wagnerite structure types   

Five commensurately modulated wagnerite structures with (2*b*), (3*b*), (5*b*), (7*b*) and (9*b*) periodicities have been reported to date (Coda *et al.*, 1967 ▶; Ren *et al.*, 2003 ▶; Chopin, Armbruster & Leyx, 2003 ▶; Armbruster *et al.*, 2008 ▶). The topological arrangement of cations and O atoms is the same in all of them. However, positional modulation of F (OH) is responsible for two distinct arc-like configurations, up (U) and down (D), in projections parallel to *c*, as emphasized for the (2*b*) model (Fig. 3 ▶). Different ordering sequences of these up (U) and down (D) arrangements lead to varying periodicities along *b* and hence the various polytypes (2*b*) (UD), (5*b*) (UDUDU), (7*b*) (UDUDUDU) and (9*b*) (UDUDUDUDU) (Chopin, Armbruster & Leyx, 2003 ▶). On the proposal of Chopin, Armbruster, Baronnet & Grew (2003 ▶), to prevent proliferation of new mineral names, the Commission on New Minerals, Nomenclature and Classification (CNMNC) of the International Mineralogical Association (IMA) has decided that wagnerite polytypes be designated by the suffixes *Ma*2*bc, Ma*5*bc, Ma*7*bc* and *Ma*9*bc* (Burke & Ferraris, 2004 ▶).

Structures of wagnerite-(5*b*) with composition (Mg_1.88_Fe_0.10_Ti_0.02_)PO_4_(F_0.61_OH_0.39_) (Ren *et al.*, 2003 ▶) and wagnerite-(9*b*) (Mg_1.97_Fe_0.03_)PO_4_(F_0.93_OH_0.07_) (Chopin, Armbruster & Leyx, 2003 ▶) were refined to reasonable residual values *R*_1_(5*b*) = 0.04 and *R*_1_(9*b*) = 0.06 in the non-centrosymmetric space group *Ia*. This showed that wagnerite structures with (5*b*) or (9*b*) periodicity have reduced symmetry, because they lose the 2_1_ axes present in the (2*b*) structure. Most surprisingly, replacement of 2% Mg by Fe in the structure of wagnerite-(9*b*) demonstrates that a small change in composition may induce a change of periodicity.

Our reinvestigation of wagnerites from over 40 localities confirms the dependence of periodicity on minor compositional variations, as will be presented below. In addition, it could be shown that the crystal structure of wagnerite may be incommensurate. Therefore, a unique superspace model for the structural description of commensurately and incommensurately modulated wagnerites was created. Of the several refined wagnerite structures using the superspace approach, five examples have been selected for discussion. The criteria for selection are the values of the **q** vectors and the intensities of satellite reflections. The results of a structural refinement on the following wagnerites will be presented: (1) a pale orange crystal from tungsten mine Panasqueira, near Fundão, Portugal (Kelly & Rye, 1979 ▶; Bussink, 1984 ▶); (2) an orange crystal from Hålsjöberg, Värmland, Sweden (Henriques, 1956 ▶); (3) an orange variety of wagnerite from Kyakhta, southern Buryatiya, Russia (Fin’ko, 1962 ▶; Izbrodin *et al.*, 2008 ▶); (4) wagnerite from Reynolds Range, Australia, drilled out of a thin section, from Vry & Cartwright (1994 ▶); (5) colourless wagnerite obtained from Webing, Austria (Kirchner, 1982 ▶). Results of the X-ray single-crystal diffraction, electron-microprobe analysis and electron microscopy of other samples of wagnerite and related minerals are listed in Table 2 ▶.

## 2. Experimental   

The experimental setting for electron-microprobe analysis of wagnerite is described by Fialin & Chopin (2006 ▶). For investigation with the electron microscope, wagnerite crystals were gently ground separately in an agate mortar under bidistilled water. When crystal fragments reached ∼ 1 µm in size, a droplet of their suspension was deposited onto a mesh copper grid coated with a 10 nm thick amorphous C film.

The high-resolution imaging and selected-area electron diffraction (SAED) patterns reported below were obtained with the Jeol 3010 high-resolution transmission electron microscope at the Centre Interdisciplinaire de Nanoscience de Marseille (CINaM) working at 300 kV and equipped with a LaB6 tip emitter, the 1.6 or 2.1 Å point-to-point pole pieces and a ± 28° double-tilt, side-entry specimen holder. In the absence of cleavage in any of the polytypes, crushing yielded thin shards and wedges with no preferred crystallographic orientation. Electrical conduction of the specimen was achieved without carbon coating. The suitable [001] zone-axis orientation was searched from pseudo-hexagonal *hk*0 diffraction patterns of the wagnerite substructure. Then the specimen was tilted slightly from this alignment to favour the contribution of satellite reflections to the Fourier summation leading to the high-resolution image contrast.

High-resolution images were typically recorded at 400–600k magnification after tuning the focusing of the objective lens under a weak-beam mode using a low-light Lhesa camera to obtain the quasi-hexagonal network of bright dots supposed to image structure channels containing F and OH. One-second film exposures were then made in full-beam mode after checking for no image drift during an increase in beam intensity. Subsequently, exposed 6 × 9 cm^2^ negative films were scanned with a Nikon Super Coolscan 8000 scanner at 4000 d.p.i. resolution to generate numerical files. Selected regions were then Fourier transformed (FT) with the NIH image/SXM software working on 2048 × 2048 matrices. The resulting frequency spectra as ‘numerical diffraction patterns’ allowed us to check beam alignment from the shape of the zeroth-order Laue zone. It also allowed further image processing when necessary through image-noise and point-defects Fourier filtering by means of inverse FT after selection of sharp spots and transmitted beam using the same program.

Single-crystal XRD was carried out on a Bruker APEX II diffractometer with Mo *K*α (0.71073 Å) X-ray radiation with 50 kV and 40 mA X-ray power. Samples were mounted on the glass needle, and measured at room-temperature conditions with 10–60 s per frame (ω-scans, scan steps 0.5 °). Data were processed using *SAINT* software (Bruker, 2011 ▶).

## 3. Results   

Table 2 ▶ lists the formula units calculated from electron-microprobe analyses of 39 samples. Difficulties concerning precise and accurate determination of fluorine contents of wagnerite and other phosphates were the subject of another study (Fialin & Chopin, 2006 ▶). Average ionic radii (Table 2 ▶) are calculated multiplying *X*_Mg_ by the radius of Mg, 0.72 Å, and (1 − *X*_Mg_) by the radius of Fe^2+^ (0.78 Å; Shannon & Prewitt, 1969 ▶), where (1 − *X*_Mg_) is the sum of the other cations (Mn, Fe, Ca and Ti).

**Figure 4:**
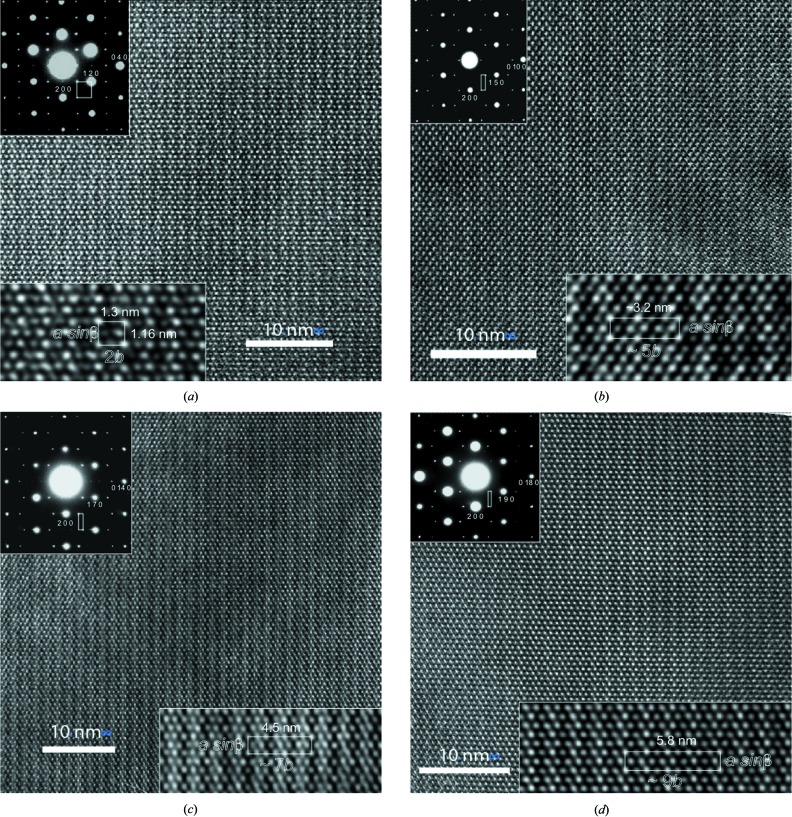
〈001〉 zone axis HRTEM micrographs of four microstructures of wagnerite. Upper left insets: SAED patterns; lower insets: zoomed views with approximate two-dimensional unit-cells as boxes. (*a*) Wagnerite (2*b*) from Miregn, Val Ambra, Lepontin Alps, Ticino, Switzerland; (*b*) wagnerite (5*b*) from Anakapalle, Andhra Pradesh, India; (*c*) wagnerite (7*b*) from Kyakhta, Russia; (*d*) wagnerite (9*b*) from Reynolds Range, Australia.

Representative samples of the ≃ (2*b*), ≃ (3*b*), ≃ (5*b*), ≃ (7*b*) and ≃ (9*b*) structures were studied by HRTEM (Figs. 4 ▶ *a*–*d*). All wagnerite polytypes are subject to electron beam damage. The phosphate grains amorphize readily in the thinnest wedges to coalescing drops lacking diffraction contrast. Substructure diffraction spots weaken concomitantly. When present, modulation fringes are better imaged in thicker regions where dynamical diffraction prevails. Given these operating conditions it is almost impossible for any polytype to record ‘structure images’ displaying all cation positions and the origin of modulation simultaneously. Instead, efforts were made to image correctly F/OH-bearing channels running along *c* only with the aim of bringing out faint contrast differences which could be indicative of differences in their content and configuration. The ‘image code’ concept (Van Tendeloo *et al.*, 1986 ▶) assumes that identical atom configurations within the unit cell display the same image at high resolution. This concept applies even if the contrast departs strongly from the local projected potential density of the structure. The latter is expected only from the thinnest regions at Scherzer underfocusing conditions of the objective lens. The modulation contrast was disappearing much quicker than the substructure contrast. This feature suggests, but does not prove, that modulation may originate from the labile F, OH sites rather than from the more stable P, *M*1 and/or *M*2 sites. Some results of electron-microscopic investigation are exemplified for different types of modulated wagnerites (Fig. 4 ▶ *a*–*d*).

The diffraction pattern of triplite appears to be pseudo-hexagonal because the strongest reflections represent the substructure in the reciprocal lattice. This feature is common to all wagnerites. Superstructure (satellite) reflections are always sharp, *i.e.* no smearing or streaking is observed. As expected, the satellite reflections are weaker than adjacent substructure reflections. Furthermore, satellite reflections are perfectly aligned along b* (no offset visible), which indicates that the modulations only occur along *b*. In (2*b*) structures, modulation spots align perfectly parallel to *a*, whereas in other ‘polytypes’, modulation spots define a zigzag ribbon resembling a string of the letter w along *a*. Each structure type has a different strongest satellite reflection along b*, namely at 2/5 corresponding to ≃ 5*b*, at 3/7 corresponding to ≃ 7*b*, or at 4/9 corresponding to ≃ 9*b*.

HRTEM images of the investigated wagnerites display strong contrast differences among the investigated members of this structural series (Figs. 4 ▶ *a*–*d*). This is consistent with the exceptional sharpness of modulation reflections (SAED patterns as upper insets in Figs. 4 ▶ *a*–*d*). After having been purposely blurred and contrasted, the blown-up raw HRTEM images (lower insets in Figs. 4 ▶ *a*–*d*) show linear patterns of bright (+) and weaker (−) dots running along *b* that mark local periodicities in that direction and from which we can draw local unit cells (lower insets in Figs. 4 ▶ *a*–*d*). As expected, these local direct-space *a*sin β − *b* unit cells correspond to the reciprocal unit cells appearing as boxes in the SAED patterns. *a*sin β is invariant for the different wagnerites, whereas *b* lengths may look at first glance to be integral multiples 2, 5, 7 and 9 of *b* of triplite.

However, there is a significant difference between (2*b*) wagnerite and the (5*b*), (7*b*) and (9*b*) wagnerites. The [+ −] motif of (2*b*) wagnerite propagates well along *b* (Fig. 4 ▶ *a*), whereas any chosen motif is progressively altered along *b* (Figs. 4 ▶ *b*–*d*) for other structures. This indicates that (2*b*) wagnerite may also be considered as commensurate, and a standard polytype of triplite. The HRTEM image contrast behaviour of other wagnerites is consistent with the incommensurability of their structures. However, it does not prove it due to the narrow field of view with constant and correct HRTEM imaging conditions that precludes long-distance commensurability to be distinguished from true incommensurability.

Owing to the location and dual intensity of light dots, a reasonable correlation may be made between + and U, − and D, *i.e.* with the arc-like arrangement of F, OH of the wagnerite structures projected along *c*. Thus, [+ −] corresponds to the [U D] sequence in (2*b*) wagnerite. For the other wagnerites, we find inside only some of the modulation fringes the following sequences or circular permutations of these, as presented in Table 3 ▶. These sequences fit with X-ray structure data for the commensurate approximation of their structure.

Analysis of sections of reciprocal space in X-ray diffraction patterns clearly showed the presence of strong parent reflections accompanied by a subset of composition-dependent ‘satellite’ reflections along b*. Using the reciprocal lattice viewer *RLATT* (Bruker, 2011 ▶), stronger reflections were separated and indexed with the *C*-centred cell corresponding to triplite [(1*b*) type] *a* ≃ 12.8, *b* ≃ 6.4, *c* ≃ 9.6, β ≃ 117°. All additional weaker satellite reflections were indexed with the **q** vector (0, β, 0) (de Wolff, 1974 ▶) using the closest main reflection along b* as reference. First-order satellite reflections found in the X-ray single-crystal diffraction pattern corresponded to strongest satellite reflections seen in SAED patterns recorded by TEM. Subsequently, data were integrated including satellite reflections using *SAINT* software (Bruker, 2011 ▶). The results are presented in Table 2 ▶. The observed systematic absences (*hklm*) *h* + *k* = 2*n* + 1, (0*k*0*m*) *m* = 2*n* + 1 and (*h*0*lm*) *l* = 2*n* + 1 unambiguously give the centrosymmetric superspace group *C*2/*c*(0β0)*s*0 (Wilson & Prince, 2004 ▶). The structure of wagnerite from Kyakhta, Russia, was solved with the software *SUPERFLIP* (Palatinus & Chapuis, 2007 ▶). This first solved structure of wagnerite was used as a parent model for structural refinements of all wagnerite crystals. Full-matrix least-squares refinement of all data sets was carried out using *JANA*2006 (Petříček *et al.*, 2006 ▶). Details on data collection and refinement for four aperiodic and one periodic (2*b*) wagnerite structures are summarized in Table 4 ▶. CIF files are provided as supporting information.^1^

## 4. Average three-dimensional structure of wagnerite   

**Figure 5:**
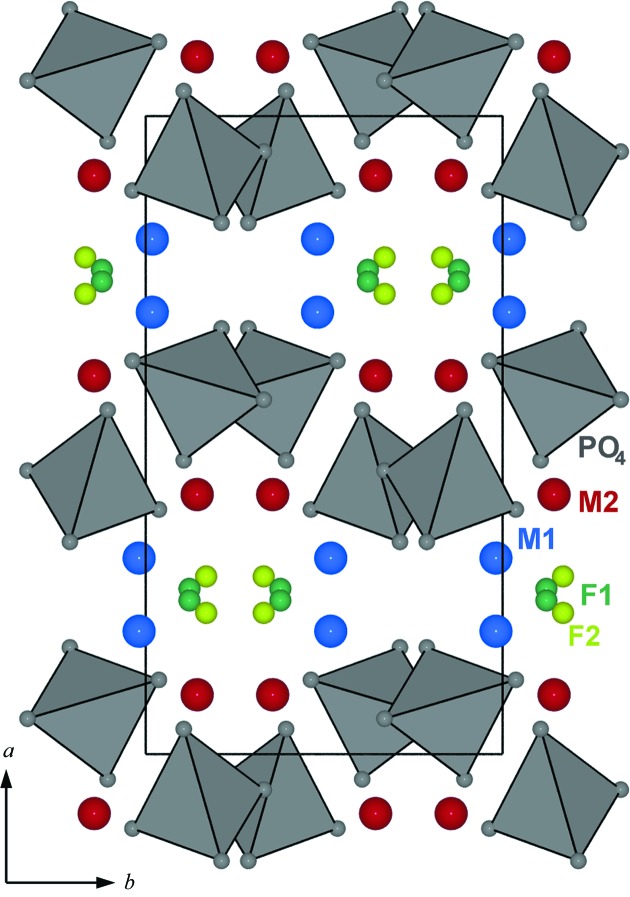
Average structure, obtained only from main reflections, of wagnerite from Khyakta in space group *C*2/*c*. PO_4_ units are displayed as grey tetrahedra, five- or six-coordinated cations as red spheres and F/O(H) as light and dark green spheres. F1 and F2 are half occupied.

To describe both periodic and aperiodic wagnerite, a unified superspace model was created using only main reflections. This model is based on an average wagnerite structure (Fig. 5 ▶) with *C*2/*c* space group and cell dimensions *a* ≃ 13, *b* ≃ 6.45, *c* ≃ 9 Å, β ≃ 117 °. The average structure has two *M* sites (*M*1 and *M*2), one P, four O and two half occupied F sites (F1 and F2) separated by *ca* 1 Å. *M*1 and *M*2 sites are fully occupied with Mg and Fe (the minor Mn is included with Fe). Depending on the arrangement of F1 and F2, both *M*1 and *M*2 are five- or six-coordinated.

*M*1 has four regular bonds to oxygen (average *M*1—O 2.07 Å) and one bond to F1 (2.11 Å) or two bonds to F2 (1.85 and 2.29 Å). *M*2 also has four regular bonds to oxygen (average *M*2—O = 2.05 Å) and one longer bond to F2 (2.19 Å) or two bonds to F1 (1.83 and 2.14 Å). The PO_4_ tetrahedra are very regular, with average bond lengths (P—O) of 1.53 Å. Thus, the average structure of wagnerite is built by two slightly distorted MO_4_F and MO_4_F_2_ polyhedra and one regular PO_4_ tetrahedron.

## 5. Superspace model   

A unified (3+1)-dimensional model includes three major parts: (1) cations: occupational and displacive modulation of Mg/Fe positions; (2) anions: occupational and displacive modulation of F or O (OH); (3) displacive modulation of the PO_4_ tetrahedron.

As in an average model, the superspace model also has two cation positions, *M*1 and *M*2. Both positions are fully occupied. These sites are hosting Mg, which according to the results of chemical analyses can be partially replaced by Fe^2+^ and Mn^2+^ and to a smaller amount by Ca and/or Ti. Considering that the scattering factors of Fe and Mn are similar for X-ray data, the amount of Fe^2+^ and Mn^2+^ are combined and treated as Fe, and the subordinate elements (Ca, Ti, Na, Al) neglected. Hence, both cation positions *M*1 and *M*2 are refined with occupational modulation. Occupational probabilities of Mg and Fe (Fe^2+^ + Mn^2+^) are constrained to be complementary. In addition, both species (Mg and Fe) at *M* sites show displacive modulation, but their coordinates, modulations and atomic displacement parameters (ADP) are constrained to be identical.

For X-ray data, the scattering power of F and O (from OH) cannot be distinguished, in particular not for mixed occupation. Consequently, these sites are refined as F or O depending on the dominant species. In an average structure two F are distributed over two half-occupied positions. In the (3+1)- dimensional model, two fluorine atoms, F1 and F2, also have two distinct positions (in *x*_1_, *x*_2_, *x*_3_), not related by symmetry operations. The alternating occupation of F1 or F2 is modelled with a crenel function (Petříček *et al.*, 1995 ▶), the results of which can adopt two distinct values only, 0 (vacancy) or 1 (occupied position). The parameters of the crenel function 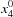 (centre of crenel function) and Δ (width of function) were refined, with the following constraints:

(1) Δ[F2] = 1 − Δ[F1];
(2) 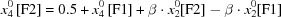.

The first constraint fixes the sum of occupancies at F1 and F2 at one. The second constraint takes care that only one F is considered in any *t*-section (real space section). In addition to occupational modulation, F sites also exhibit positional modulation. A Legendre polynomial is used to combine the crenel function with positional modulation (Dušek *et al.*, 2010 ▶). For all other sites (one P and four O), the modulation of positional and anisotropic displacement parameters was refined with harmonic functions. The sine and cosine terms of up to the third harmonic wave of the modulation functions may be used, depending on the highest observed order of satellites and their number and intensity. In addition, depending on chemical composition (*e.g.* concentration of OH groups in the anionic part) and data quality, H positions could be found in difference Fourier maps. Four modulated structures of wagnerite will be presented. Figures of *t*-plots and Fourier maps are only shown for wagnerite from Kyakhta. The type and degree of modulation in four additional samples will be described. Selected bond distances, including average (average) and extreme (minimum and maximum), caused by modulation in the structures of different wagnerites are given in Tables 5–11 of the supporting information. In all investigated wagnerite structures, the PO_4_ tetrahedron behaves almost as a rigid unit, just tilting a little bit around its centre of gravity. Thus, small variations of the average P—O bonds will be briefly discussed.

### 5.1. Wagnerite from Kyakhta, Russia (orange variety)   

**Figure 6:**
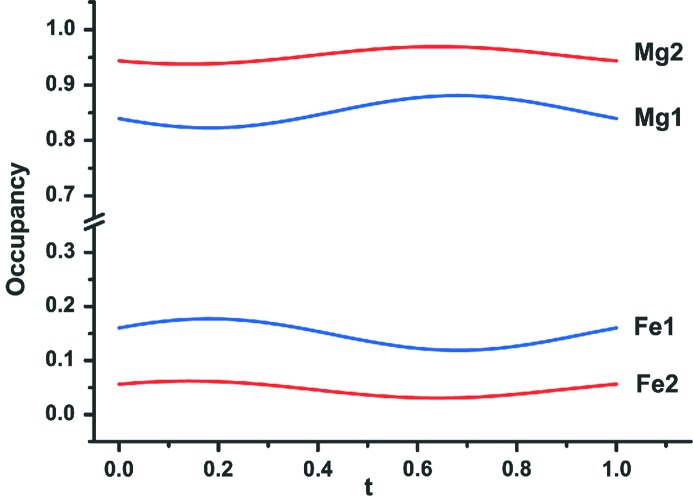
Occupational modulations of Mg and Fe atoms on *M*1 and *M*2 sites in wagnerite from Kyakhta: *occ* (*M*1) = *occ* (Mg1) + *occ* (Fe1) and *occ* (*M*2) = *occ*(Mg2) + *occ* (Fe2).

**Figure 7:**
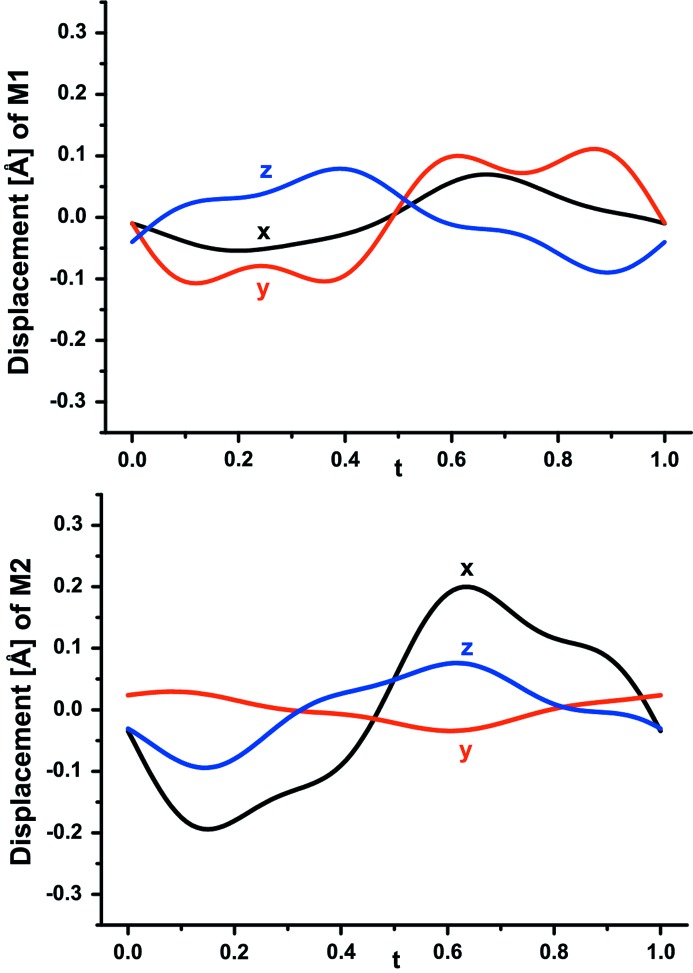
Displacive modulation of cations on *M*1 and *M*2 sites in wagnerite from Kyakhta as a function of *t*.

**Figure 8:**
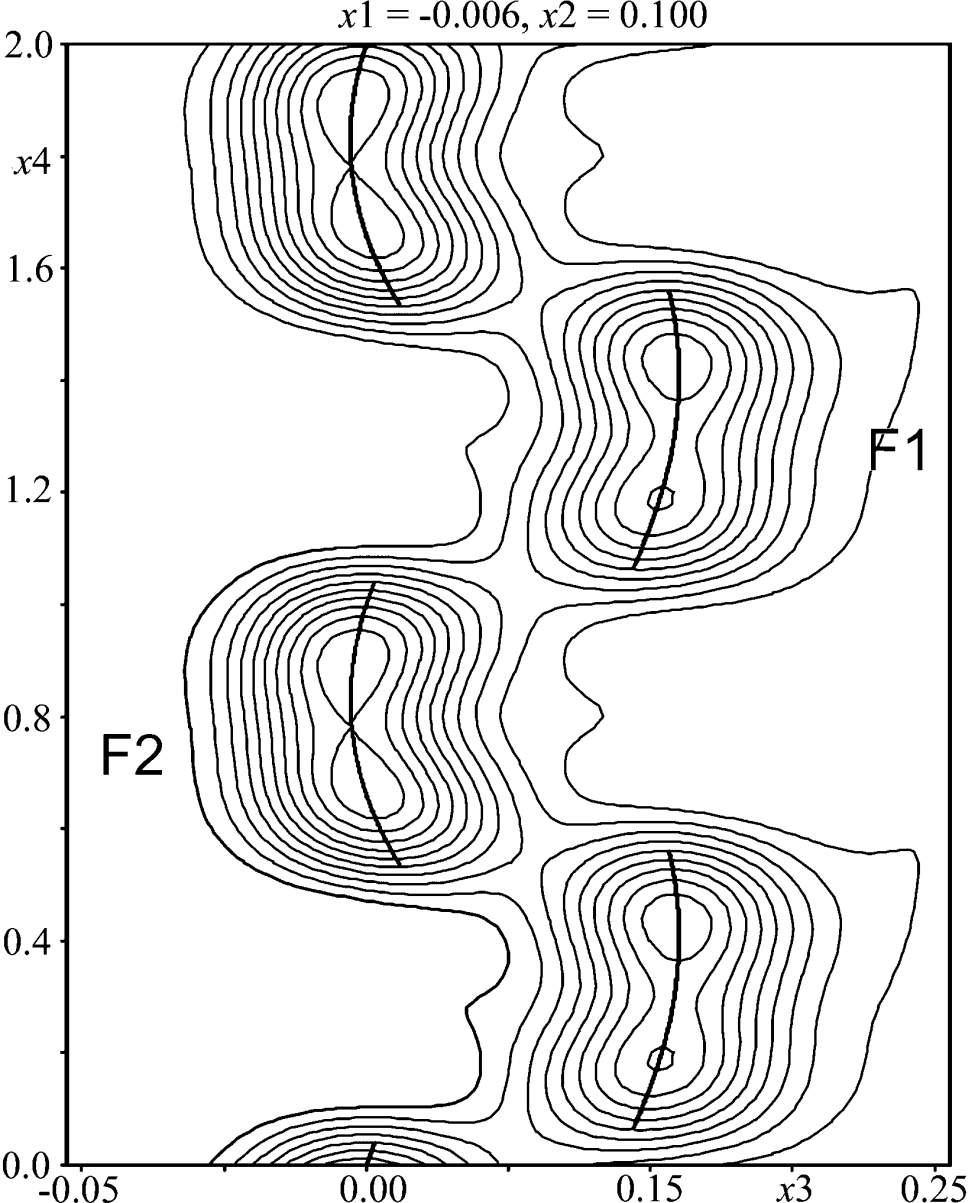
The crenel function modulation of F1 and F2. *x*_3_ − *x*_4_ map intersecting the four-dimensional *F*_obs_ Fourier synthesis at *x*_1_ = 0.006 and *x*_2_ = 0.100.

**Figure 9:**
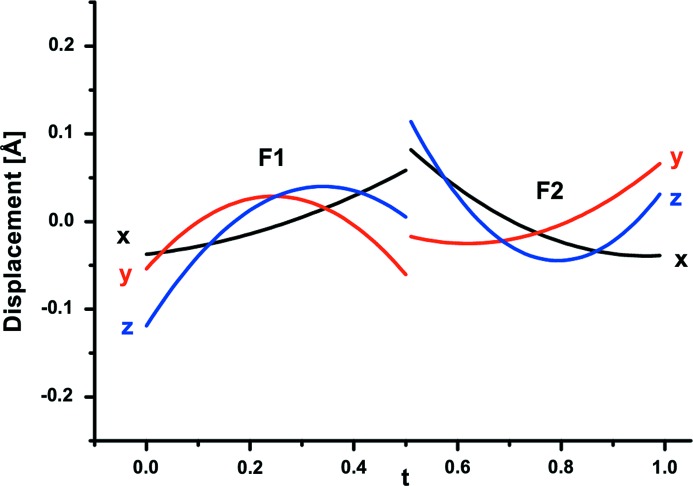
Displacive modulations of F(O) in wagnerite from Kyakhta in 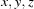 displacement as a function of *t*.

**Figure 10:**
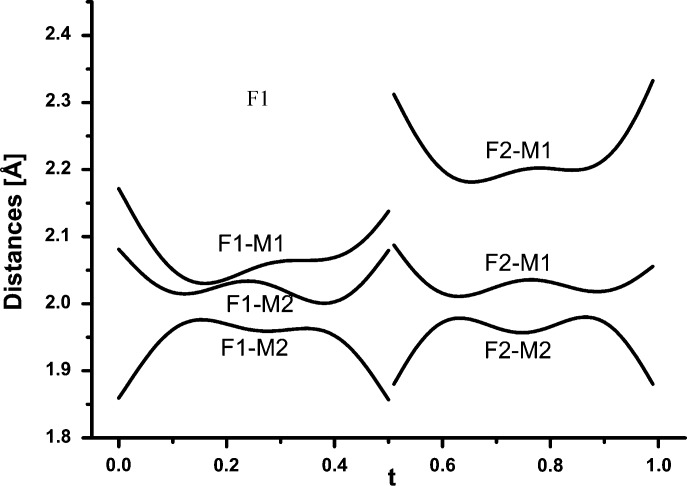
Coordination of F presented as a plot of bond lengths to *M* sites as a function of *t* in wagnerite from Kyakhta.

Refinement of the structure was based on all main and satellite reflections up to third order (Table 4 ▶). Following the above-described recipe, occupational probabilities of Mg and Fe^2+^ (Fe^2+^ + Mn^2+^) are refined complementarily and they are presented as a function of the internal coordinate *t* (Fig. 6 ▶). The Fe content at *M*1 varies with modulation from 12 to 18% and at *M*2 between 3 and 6%. The average composition of the *M*1 + *M*2 sites, 90% Mg and 10% (Fe^2+^ + Mn^2+^) is very close to the average obtained by electron-microprobe analysis (Table 2 ▶). In addition, both *M* sites exhibit displacive modulation apparent in corresponding Fourier maps (Fig. 7 ▶). The modulation of *M*1 is more pronounced along *x*_2_ (b*) and of *M*2 along *x*_1_ (a*). The occupation of F is refined with a crenel function (Fig. 8 ▶). The refined value of Δ = 0.5039 (9) indicates that F is equally distributed over two positions. In addition, F1 and F2 show significant displacive modulation in all three directions (Fig. 9 ▶). A plot of interatomic distances as a function of *t* confirms that F1 and F2 are always threefold-coordinated by *M*1 and *M*2 (Fig. 10 ▶). F1 has three bonds to *M* sites, F1—*M*1 = 2.028 (3) Å (average) and F1—*M*2 = 2.0736 (17) Å and an additional F1—*M*2 = 1.941 (3) Å (average). F2 has two bonds to *M*1 [1.955 (2) and 2.221 (2) Å (average)] and one to *M*2 [2.030 (3) Å (average)].

**Figure 11:**
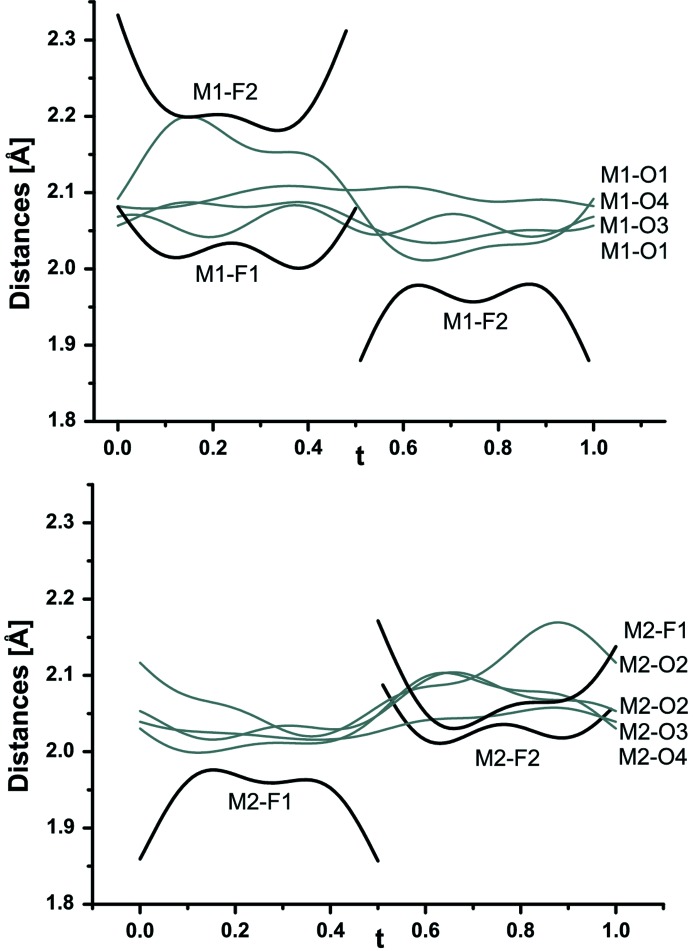
Coordination of *M*1 and *M*2 atoms with four O and one or two bonds to F presented as the dependence of bond lengths as a function of *t* in wagnerite from Kyakhta.

**Figure 12:**
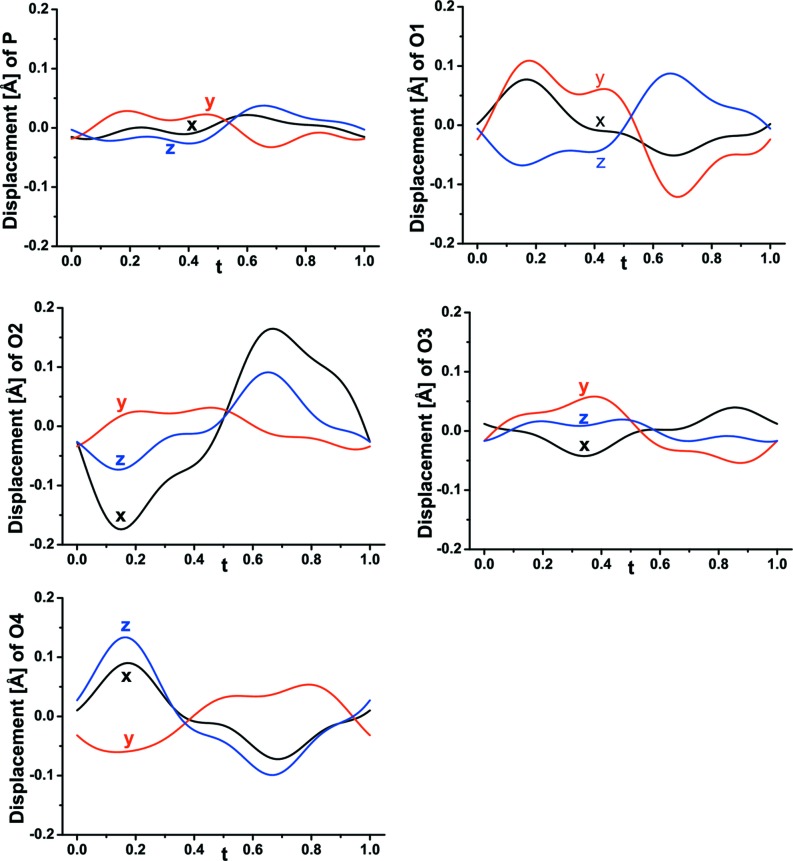
Displacive modulation of atoms in PO_4_ units as a function of *t* in wagnerite from Kyakhta.

The coordination of *M*1 and *M*2 is displayed in Fig. 11 ▶ and Table 7 of the supporting information. In sections from *t* = 0 to *t* = 0.5, *M*1 is six- coordinated with four regular bonds to O and one to F1 [average 2.027 (3)–2.155 (3) Å] and one longer bond to F2 [average 2.221 (2) Å]. Therefore, *M*2 is five-coordinated with four O atoms [average 2.012 (3)–2.053 (3) Å] and one shorter bond to F2 [average 1.943 (3) Å]. Between *t* = 0.5 and *t* = 1, the situation is reversed. *M*1 is five-coordinated with four O atoms [average 2.037 (3)–2.096 (3) Å] and a shorter bond to F2 [average 1.955 (2) Å]. *M*2 has regular sixfold coordination *M*_2_O_4_F_2_ [average 2.030 (3)–2.118 (2) Å]. In Figs. 12 ▶(*a*)–(*e*) the positional modulation of the PO_4_ tetrahedron is displayed. The *t*-plots suggest a very small displacive modulation of P associated with displacement of the pairs O1/O4 and O2/O3. The biggest displacive modulation is found for O2 connecting the PO_4_ tetrahedron with *M*1 and *M*2 polyhedra. Nevertheless, the tetrahedron preserves average P—O distances between 1.533 (2) and 1.540 (3) Å (Table 7 of the supporting information).

In addition, the final difference-Fourier map indicated (residual peak of 0.7 e) the position of partly occupied H close to F1, which represents in this case an O site (OH group).

### 5.2. Wagnerite from Panasqueira, Portugal   

Refinement of the structure was based on all the main and first-order satellite reflections (Table 4 ▶). Refined occupational probabilities of Mg and Fe^2+^ (Fe^2+^ + Mn^2+^) converged to 29–33% Mg at *M*1 and to 51–71% Mg at *M*2, as well as to 67–71% of (Fe^2+^ + Mn^2+^) at *M*1 and to 29–48% at *M*2. The average composition of *M* cations of 46% Mg and 54% (Fe^2+^ + Mn^2+^) agrees fairly well with the results (40% Mg) of electron-microprobe analysis (Table 2 ▶). The obtained value of ΔF1 = 0.5303 (3) in the crenel occupation function indicates that F slightly prefers F1 over F2. This has consequences on the *M*1 and *M*2 coordination (Table 5 of the supporting information). Between *t* = 0 and *t* = 0.53, *M*1 has five regular bonds to four O and to one F [average 2.085 (11)–2.156 (1) Å]. If F2 is occupied (from *t* = 0 to *t* = 0.47) one additional longer bond to F2 [average 2.324 (8) Å] exists. In the section between *t* = 0.53 and *t* = 1, *M*1O_4_F_2_ has five average bonds between 2.034 (11) and 2.1431 (10) Å. Between *t* = 0 and *t* = 0.53, *M*2 has five regular bonds, comprising 4 × O and F1 [average 1986 (6) Å]. In the sections from *t* = 0.53 to *t* = 1, the *M*2O_4_F1F2 polyhedron has six bonds between (average) 1.925 (9) and (average) 2.181 (4) Å. The PO_4_ tetrahedron shows more pronounced tilting than in the structure of Kyakhta wagnerite. All average P—O bonds are between 1.5314 (11) and 1.5424 (14) Å (Table 5 of the supporting information).

### 5.3. Wagnerite from Hålsjöberg, Sweden   

From X-ray data of wagnerite from Hålsjöberg, Sweden, up to the third-order satellite reflections are visible (Table 4 ▶). Statistically around 10% of second- and third-order reflections were observed, but their intensity was weak. Thus, the refinement was performed with all main reflections and first-order satellites only. Site populations of 52–60% Mg and 40–48% of (Fe^2+^ + Mn^2+^) were refined at *M*1 and 72–86% Mg and 14–28% (Fe^2+^ + Mn^2+^) at *M*2. The average composition at *M* sites (69% Mg and 31% Fe + Mn) is close to the one obtained by electron-microprobe analysis: 64% Mg, 22% Mn and 11% Fe (Table 2 ▶). The width of the crenel function at F1 [Δ = 0.504 (1)] shows a minor preference of F for this position. *M*1 and *M*2 are each to 50%, five- and six-coordinated (Table 6 of the supporting information). Between *t* = 0 and *t* = 0.5, *M*1 has five bonds to O and F1 [average 2.072 (7) to 2.166 (1) Å] and one longer bond to F2 [average 2.241 (5) Å]. *M*2 has regular fivefold coordination (*M*2O_4_F1) with average bonds [average 1.948 (7)–2.068 (1)  Å]. For sections from *t* = 0.5 to *t* = 0.1, both polyhedra around *M*1 and *M*2 have regular coordination, *M*1O_4_F1 [average 1.978 (7)–2.1204 (10) Å] and *M*2O_4_F1F2 [average 2.032 (8)–2.1298 (10) Å]. The PO_4_ tetrahedron shows the same behaviour as in other wagnerite structures, with P—O bond lengths (average) between 1.5334 (10) and 1.5415 (13) Å (Table 6 of the supporting information).

### 5.4. Wagnerite from Reynolds Range, Australia   

Structure refinement of the wagnerite from Reynolds Range was based on all main and first-order satellite reflections (Table 4 ▶). The chemical composition of the investigated crystal was close to the Mg wagnerite end-member (Table 2 ▶). Population refinements in our superspace model confirmed this composition. Occupational probabilities of (Fe^2+^ + Mn^2+^) at *M*1 are 2.5–4% and 0–1% at *M*2. The average Fe + Mn content of 2% confirms the results of the microprobe analysis (Table 2 ▶). F is perfectly distributed over two positions [ΔF1 = 0.5016 (7)]. For *t* = 0 up to *t* = 0.5, *M*1 has five bonds to O and F1 [average 2.061 (2)–2.151 (1) Å] and one slightly longer bond to F2 [average 2.2154 (2) Å]. The *M*2O_4_F1 polyhedron has five average bonds between 1.938 (2) and 2.051 (2) Å. Between *t* = 0.5 and *t* = 1, the *M*1O_4_F1 polyhedron has average bonds between 1.940 (2) and 2.087 (2) Å, and the *M*2O_4_F1F2 octahedron from (average) 2.044 (2) to 2.1113 (17) Å (Table 8). The PO_4_ tetrahedron behaves as rigid unit with the average bonds from 1.5328(17) to 1.538(2) Å (Table 8 of the supporting information).

### 5.5. Wagnerite from Webing, Austria   

Of the structures presented in this paper, only that of wagnerite from Webing, Austria, is periodic. Based on chemical analysis (Table 2 ▶) this sample can be considered as the end-member Mg_2_(PO_4_)F. Results of refinements both with a periodic supercell (in *P*2_1_/*n* space group with 2*b* parameter) or with superspace formalism [*C*2/*c*(0β0)*s*0 with **q** = *0.5* b*] are deposited to allow easy comparison with other (3)- or (3+1)-dimensional structures. Selected bond distances for both models are presented in Tables 9–11 of the supporting information.

Structure refinement in the superspace group *C*2/*c*(0β0)*s*0 with **q** = 0.5b* was based on all main and first-order satellite reflections (Table 4 ▶). There were no correlations larger than 0.7 in the last refinement cycle. Corresponding to chemical analysis (Table 2 ▶), *M*1 and *M*2 positions are fully occupied by Mg. F is perfectly distributed over two positions, for which only sine terms of the harmonic wave of the positional and ADP modulation function are refined. For the remaining atoms, two Mg, one P and four O, both sine and cosine terms of the positional and ADP modulation function were refined. As in the above described aperiodic structures, Mg1 and Mg2 atoms are five- or six-coordinated, depending on the position of F (Table 9 of the supporting information). The average bonds for five- and six-coordinated Mg1 are between 1.9422 (7) and 2.2411 (5) Å and for Mg2 between 1.9371 (4) and 2.0813 (4) Å. The PO_4_ tetrahedron corresponds to those in other wagnerite structures, with all bonds between 1.5284 (4) and 1.5464 (4) Å (Table 9 of the supporting information).

Using the supercell formalism a structure refinement was performed in space group *P*2_1_/*n* with a doubled *b* parameter (Table 2 ▶). In this structure four Mg sites correspond to *M*2 and four additional sites to *M*1. Out of four *M*1 polyhedra, two have regular sixfold and two fivefold coordination. *M*—O/F bond distances vary between 1.9414 (5) and 2.2394 (4) Å (Table 10 of the supporting information). All P—O bond lengths are in the range between 1.5255 (3) and 1.5474 (4) Å (Table 11 of the supporting information). One difference between the two refinement strategies is a small deviation in unit-cell parameters (Tables 2 ▶ and 4 ▶) as a consequence of differences in the way reflections are integrated.

## 6. Discussion   

There are many examples of minerals having modulated structures that give satellite reflections observable with electron diffraction, but only a few of them have been studied with superstructure formalism (Bindi, 2008 ▶, and references therein). It is unusual to find minerals giving satellite reflections which are sufficiently strong and sharp enough for structural refinement.

Our investigation shows that most wagnerite samples have modulated structures. Therefore, in refining the average structure, information provided by the satellite reflections is being deliberately neglected. Another approach to handling such structures is to discard any differences between the main and satellite reflections and to treat all reflections equally, that is, the structure is refined in a supercell with pseudo-commensurate periodicity and all observed satellite reflections indexed. Such an approach is successful if satellite reflections are commensurate, as described in the (5*b*) model by Ren *et al.* (2003 ▶). If the structure is incommensurate, satellite reflections do not fit the grid of the supercell lattice and cause poor agreement factors, large standard deviations, split atom positions and large ADP. The β components of the modulation vectors **q** = βb* for four wagnerite samples discussed in this paper are close to commensurate values, especially with ‘larger cells’ [*e.g.* β = 0.34599 (3) ≃ 1/3; β = 0.41066 (3) ≃ 2/5 (0.4); β = 0.427560 (18) ≃ 3/7 (0.42857) and β = 0.44652 (2) ≃ 4/9 (0.4444)]. Therefore, it is not surprising that refinements using superstructure models can also provide reasonable results. However, this refinement strategy entails additional difficulties and problems, as discussed below.

In a refinement of Kyakhta wagnerite with a primitive lattice (space group *P*2_1_) and sevenfold supercell, there are 56 symmetry-independent *M* sites, 28 P sites, 112 O and 28 F sites. Simple refinement of atomic coordinates and isotropic displacement parameters, restricted to species, gives a total of 710 parameters, with large correlations among them. In contrast, using a superspace approach for such a commensurate 7*b* cell, only 166 parameters are needed for the refinement of nine atom sites (two *M*, one P, four O and two F) and their positional, occupational and anisotropic displacement parameters. Thus, a superspace approach is an efficient tool for dealing with commensurate structures with large unit cells.

Commensurate and incommensurate structures of wagnerite Mg_2 − *x*_(Fe, Mn, Ca, Ti…)_*x*_(PO_4_)(F, OH, O) may be considered products of a structural branching process, *i.e.* increasing complexity of structural modulation with solid solution in which the (1*b*) and (2*b*) structure types function as end-members. The modulation complexity is related to a chemical complexity due to different compositions of the various (1*b*) and (2*b*) end-members shown in the two triangular diagrams in Fig. 1 ▶.

**Figure 13:**
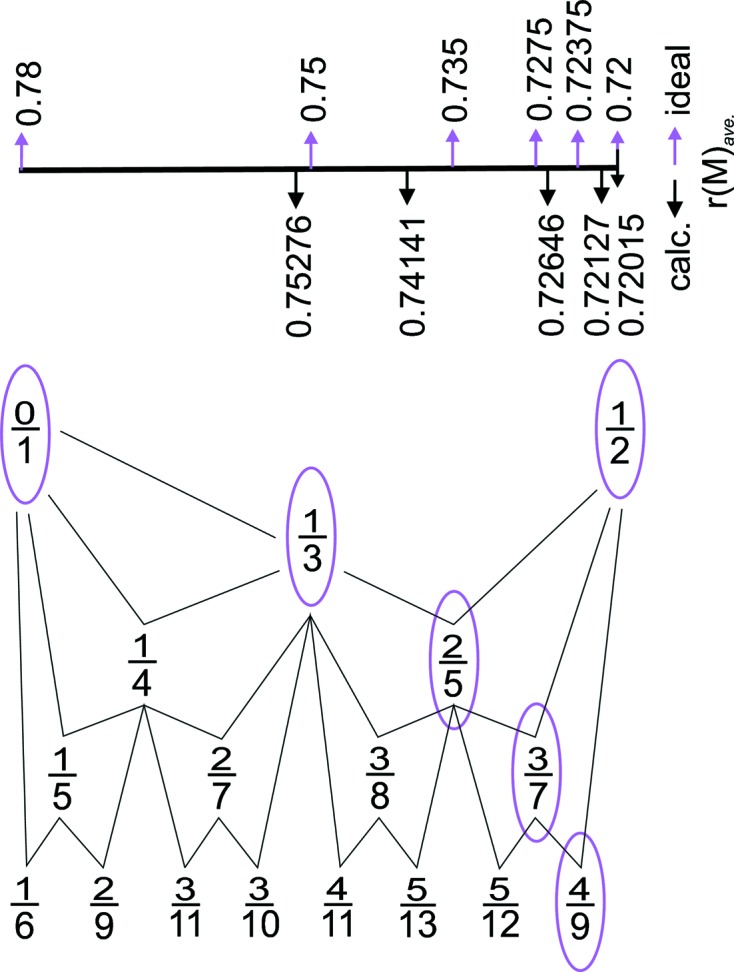
Farey tree (Hardy & Wright, 2003 ▶). The marked branches correspond to the values of the main satellite reflections observed in the crystals studied by us. The corresponding average ionic radii calculated for *M* sites are presented on the scale: ideal values above and calculated values for our five selected wagnerite crystals below (see text).

This is confirmed by the average structure model with (1*b*) cell dimensions as for triplite and F distribution conforming to the distributions in both the (1*b*) and (2*b*) types. Wagnerite structures with a (5*b*) (UDUDU), (7*b*) (UDUDUDU) and (9*b*) (UDUDUDUDU) cell could be considered as structures with the faults in which the (2*b*) (UD) periodicity is violated on every fifth, seventh and ninth sequence of the structure. Another indicator for the suggested branching process is that rational β values for observed modulation vectors (**q** = *β* b*) are very close to the branches of Farey tree series (Hardy & Wright, 2003 ▶). Generating Farey medians successively between 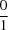 and 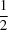, the obtained values are 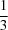, 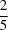, 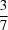, 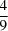 *etc*. These values correspond to the strongest satellite reflections along b* observed in different wagnerite samples by HRTEM: 2/5 in the ≃ (5*b*) structure, 3/7 in ≃ (7*b*) and 4/9 in ≃ (9*b*) type. Each branch of a Farey tree has two ‘parents’ in the level above, *e.g.* 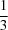 is a ‘child’ of 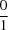 and 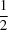 or 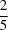 is a ‘child’ of 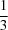 and 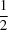. In wagnerites, this parent–child relationship is associated with chemical composition, because the value of the modulation vector or branch of the Farey tree can be predicted from the calculated average cation radius on the *M* position (Fig. 13 ▶). For the 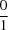 branch [(1*b*) structure type] let us consider pure Fe_2_(PO_4_)F, with a cation radius of 0.78 Å and for the 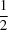 branch [(2*b*) structure type], Mg_2_(PO_4_)F or Mg_2_(PO_4_)OH with cation radius 0.72 Å. The average value of the *M* radius for the child structure should be between the values of the parent structures. For simplicity, only parameters for sixfold coordination are calculated (Shannon & Prewitt, 1969 ▶), and the cation composition is restricted to only two species, Mg (radius 0.72 Å) and Fe^2+^ (radius 0.78 Å), where the latter also accounts for minor Mn^2+^. Therefore, for the 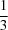 branch the predicted radius at *M* is 0.75 Å, for 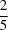, 0.735 Å, for 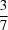, 0.7275 Å and for 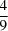, 0.72375 Å (Fig. 13 ▶), values in reasonable agreement with the corresponding average ionic radii determined for our selected wagnerite crystals, respectively, 0.7528 Å [≃ (3*b*), Panasqueira], 0.7414 Å [≃ (5*b*), Hålsjöberg], 0.7275 (≃ (7*b*), orange Kyakhta] and 0.7213 [≃ (9*b*), Reynolds Range] (Table 2 ▶). In summary, the Farey tree series with average ionic radius shows a remarkable qualitative resemblance with the observed modulation in wagnerite and may be used as a simplified approach to explain complex crystal-chemical relationships. In actuality, we expect that the relation between modulation and *M*-site chemistry is more complex. The different periodicity along b* of wolfeite Fe_2_(PO_4_)(OH) and zwieselite Fe_2_(PO_4_)F indicates that the OH → F substitution influences the modulation. In addition, the modulation is sensitive to whether the average *M* ionic radius is increased by Fe^2+^ or Mn^2+^ (radius 0.82 Å). Lastly, the pressure–temperature conditions under which wagnerite crystallized and was annealed could affect the modulation, *e.g.* Fe^2+^ and Mn^2+^ should become more disordered with increasing temperature.

Modelling the structure of wagnerite, with a (3+1)-dimensional approach in which F/OH is subject to occupational and displacive modulation appears justified, particularly when we compare bonds and coordination polyhedra around *M* sites. In all selected wagnerite structures, both sites *M*1 and *M*2 are partially five or six coordinated, but mean bond lengths and angles are in very good agreement with expected values for non-modulated structures (Allen *et al.*, 2006 ▶).

## 7. Conclusion   

The unified superspace model for the structural description of periodically and aperiodically modulated wagnerite is created with occupational and displacive modulations of Mg/Fe atoms, occupational and displacive modulation of F (O) atoms and displacive modulation of the PO_4_ tetrahedron.

The superspace model is superior to ‘average cell’ and ‘supercell’ models because: (1) periodic and aperiodic wagnerite structures can be refined with a common space group; (2) it enables refinement of positional and occupational modulation of atoms, which is essential for this structure type; (3) it simplifies the description of positional and occupational modulation of Mg/Fe and F/OH, and their connectivity; (4) it converges to better residual values with a lower number of refined parameters and less correlation among parameters.

## Supplementary Material

Crystal structure: contains datablock(s) wagnerite_3+1, publication_text, I, 3b, 5b, 7b, 9b, 2b. DOI: 10.1107/S2052520613031247/dk5018sup1.cif

Structure factors: contains datablock(s) I. DOI: 10.1107/S2052520613031247/dk5018Isup2.hkl

Structure factors: contains datablock(s) 3b. DOI: 10.1107/S2052520613031247/dk50183bsup3.hkl

Structure factors: contains datablock(s) 5b. DOI: 10.1107/S2052520613031247/dk50185bsup4.hkl

Structure factors: contains datablock(s) 7b. DOI: 10.1107/S2052520613031247/dk50187bsup5.hkl

Structure factors: contains datablock(s) 9b. DOI: 10.1107/S2052520613031247/dk50189bsup6.hkl

Structure factors: contains datablock(s) 2b. DOI: 10.1107/S2052520613031247/dk50182bsup7.hkl

Tables 5-11 with selected bond distances. DOI: 10.1107/S2052520613031247/dk5018sup8.pdf

8712E0W5yP

CCDC references: 971920, 971921, 971922, 971923, 971924, 971925

## Figures and Tables

**Table 1:** Synthetic and natural end-members, with unit-cell dimensions in unified setting

	Space group						
Compound	Rep.	Transf.	*a* ()	*b* ()	*c* ()	()	*V* (^3^)
(1) Mg_2_(PO_4_)F†	*P*2_1_/*n*	*P*2_1_/*n*	12.7631 (4)	12.6565 (4)	9.6348 (3)	117.5954 (11)	1379.32
(2) Fe_2_(PO_4_)F	*I*112/*a*	*C*2/*c*	13.0211 (39)	6.4890 (10)	9.8900 (30)	118.624 (20)	733.52
(3) Mn_2_(PO_4_)F	*C*2/*c*	*C*2/*c*	13.4100 (40)	6.5096 (5)	10.0940 (20)	119.990 (10)	763.17
(4) Mg_2_(PO_4_)OH	*P*2_1_/*c*	*P*2_1_/*n*	12.8445 (55)	12.8590 (30)	9.6560 (10)	116.986 (26)	1421.21
(5) Fe_2_(PO_4_)OH	*P*2_1_/*a*	*P*2_1_/*n*	12.9983 (17)	13.1970 (10)	9.7385 (9)	116.601 (8)	1493.69
(6) (Mn,Fe)_2_(PO_4_)OH†	*P*2_1_/*a*	*P*2_1_/*n*	13.2232	13.2760	9.9430	117.347	1550.42

Matrices for transformation of reported cells: *P*2_1_/*a* 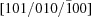 *P*2_1_/*c* 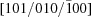 *P*2_1_/*n*; *I*112/*a* *I*12/*a*1 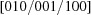 *C*2/*c* 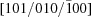. Source of samples: (1) this paper (wagnerite from Webing); (2) Yakubovich *et al.* (1978 ▶); (3) Rea Kostiner (1972 ▶); (4) Raade Rmming (1986 ▶); (5) Hatert (2007 ▶); (6) Waldrop (1968 ▶).

† Natural; synthetic samples have not been reported.

**Table 2:** Results of X-ray single-crystal diffraction and electron-microprobe analysis for wagnerite and a few related minerals from different localities Chemical compositions are presented for *M* and (F, OH) positions in *M*_2_(PO_4_)(F, OH), where *X* is mole fraction. The average ionic radii for *M* is calculated as *r*(*M*) (average) = *X*_Mg_(0.72)+(1*X*_Mg_)(0.78), parameters from Shannon (1976 ▶).

No.	Origin of the sample	*a* ()	*b* ()	*c* ()	()	**q** = b*,	Period.	*r*(*M*)_ave._	*X*_Mg_	*X*_Fe_	*X*_Mn_	*X*_Ca_	*X*_Ti_	*X*_Na_	*X*_Al_	*X*_F_
(1)	Reynolds Range, Australia	12.7707(2)	6.33940(10)	9.64620(10)	117.5242(5)	0.44652(2)		0.72127	0.979	0.016	0.001	0.003	0.002	0.000	0.000	0.98
(2)	In Ouzzal, NW Hoggar, Algeria	12.7758(2)	6.3378(1)	9.6480(2)	117.5720(5)	0.44513(3)		0.72307	0.949	0.032	0.001	0.001	0.017	0.000	0.000	0.84
(3)	Kyakhta, Russia; orange	12.7978(2)	6.35230(10)	9.66420(10)	117.5670(10)	0.427560(18)		0.72646	0.892	0.078	0.019	0.001	0.010	0.000	0.000	0.87
(4)	Kyakhta, Russia; yellow	12.8018(15)	6.3488(7)	9.6787(11)	117.739(3)	0.39120(2)		0.72902	0.850	0.124	0.024	0.001	0.001	0.000	0.000	1.00
(5)	Skinov, Czech Republic	12.7580(3)	6.3332(2)	9.6421(2)	117.5600(11)	0.40927(3)		0.72149	0.975	0.019	0.000	0.004	0.002	0.000	0.000	1.00
(6)	Mont. St Hilaire, Canada	12.7667(3)	6.3359(1)	9.6486(2)	117.5951(7)	0.44961(2)		0.72235	0.961	0.034	0.004	0.000	0.000	0.000	0.000	0.98
(7)	Chelyabinsk, S. Urals, Russia	12.771(3)	6.332(1)	9.654(1)	117.63(2)	†	(5*b*)	0.72298	0.950	0.030	0.008	0.008	0.004	0.000	0.000	0.97
(8)	Benson Mine, New York, USA	12.8211(2)	6.35612(9)	9.6975(1)	117.7865(7)	0.39026(4)		0.73225	0.796	0.142	0.060	0.001	0.001	0.000	0.000	0.95
(9)	Mount Pardoe, Antarctica	12.7640(2)	6.3322(1)	9.6434(1)	117.5895(7)	0.40435(4)		0.72293	0.951	0.040	0.001	0.001	0.006	0.000	0.000	0.93
(10)	Anakapalle, India	12.7676(2)	6.33236(8)	9.6472(1)	117.5707(5)	0.40600(5)		0.72177	0.970	0.019	0.000	0.006	0.004	0.000	0.000	0.93
(11)	Karasu, Kyrgyzstan	13.026(3)	6.429(1)	9.853(15)	118.46(14)	†	(5*b*)	0.76103	0.316	0.379	0.283	0.009	0.012	0.000	0.000	0.82
(12)	Sierra Albarrana, Spain	12.908(2)	6.398(1)	9.7636(8)	117.948(10)	†	(5*b*)	0.74738	0.544	0.320	0.118	0.005	0.013	0.000	0.000	0.80
(13)	Kyrk-Bulakh, Kyrgyzstan	12.9769(8)	6.4340(4)	9.8119(6)	117.9842(8)	0.40741(6)		0.75347	0.442	0.366	0.170	0.004	0.017	0.000	0.000	0.74
(14)	Hlsjberg, Sweden	12.8840(2)	6.38890(10)	9.37840(10)	117.7994(4)	0.41066(3)		0.74141	0.643	0.222	0.112	0.002	0.019	0.001	0.000	0.72
(15)	Albres, France	12.9462(7)	6.4378(4)	9.7957(5)	117.8892(12)	0.40906(4)		0.75430	0.428	0.426	0.136	0.003	0.007	0.000	0.000	0.72
(16)	Tsaobismund, Namibia	13.0731(2)	6.4513(1)	9.8789(1)	118.5113(6)	0.38714(6)		0.76389	0.268	0.396	0.320	0.010	0.004	0.001	0.000	0.79
(17)	Cap de Creus, Spain	12.9389(4)	6.4224(2)	9.7765(3)	117.861(1)	0.4193(1)		0.75229	0.462	0.377	0.126	0.003	0.033	0.000	0.000	0.60
(18)‡	Larsemann Hills, Antarctica	12.766(4)	6.332(6)	9.645(2)	117.589(3)	0.40000		0.74311	0.916	0.085	0.002	0.002	0.018	0.000	0.000	0.61
(19)	Himachal Himalaya, India	12.9242(7)	6.4122(3)	9.7716(5)	117.881(2)	0.40928(9)		0.74859	0.507	0.365	0.102	0.004	0.020	0.000	0.000	0.74
(20)	Salamanca, Spain	13.0312(3)	6.4478(1)	9.8444(2)	118.2198(10)	0.40426(3)		0.76138	0.310	0.385	0.283	0.006	0.015	0.001	0.000	0.68
(21)	Webing, Austria	12.7633(4)	6.3282(2)	9.6350(3)	117.5985(11)	0.50000		0.72015	0.998	0.000	0.002	0.000	0.000	0.000	0.000	0.97
(22)	Reyershausen, Germany	12.7526(16)	6.3284(6)	9.6359(3)	117.553(6)	†	(2*b*)	0.72027	0.996	0.000	0.000	0.002	0.000	0.000	0.002	0.94
(23)	Santa Fe Mountains, USA	12.7783(9)	6.3410(5)	9.6494(7)	117.5278(9)	0.49841(5)		0.72236	0.961	0.023	0.013	0.001	0.002	0.000	0.000	0.89
(24)	Tonagh Island, Antarctica	12.9084(17)	6.398(1)	9.7636(8)	117.988(10)	†	(2*b*)	0.72306	0.949	0.036	0.001	0.001	0.013	0.000	0.000	0.89
(25)	Christmas Point, Antarctica	12.7821(6)	6.3469(3)	9.6563(4)	117.5319(14)	0.46734(5)		0.72447	0.926	0.064	0.002	0.001	0.007	0.000	0.000	0.83
(26)‡	Werfen, Austria	12.819(11)	6.3395(80)	9.644(7)	117.4411(11)	0.5000		0.72244	0.959	0.036	0.003	0.001	0.000	0.000	0.000	0.83
(27)‡	Hllgraben, Austria	12.7694(2)	6.33423(1)	9.6365(1)	117.4808(6)	0.49914(3)		0.72065	0.989	0.001	0.000	0.001	0.008	0.000	0.000	0.78
(28)	Bamble, Norway	12.7797(9)	6.3417(4)	9.6428(7)	117.5152(9)	0.49822(5)		0.72063	0.989	0.001	0.000	0.001	0.008	0.000	0.000	0.78
(29)	Miregn, Lepontin Alps, Switzerland	12.8112(3)	6.3700(7)	9.6630(20)	117.384(4)	0.4990(11)		0.72514	0.914	0.071	0.011	0.001	0.002	0.000	0.000	0.67
(30)	Mount Painter, Australia	12.7957(2)	6.3590(1)	9.6510(1)	117.3995(6)	0.49957(4)		0.72254	0.958	0.028	0.010	0.001	0.003	0.000	0.000	0.67
(31)	Star Lake, Manitoba, Canada	12.809(2)	6.366(1)	9.665(2)	117.381(4)	0.4838(3)		0.72480	0.920	0.065	0.001	0.001	0.013	0.000	0.000	0.65
(32)	Panasqueira, Portugal	13.0183(2)	6.41490(10)	9.84110(10)	118.5620(10)	0.34599(3)		0.75276	0.402	0.228	0.366	0.000	0.000	0.000	0.000	0.78
(33)‡	OH-wagnerite, Dora Maira, Italy	12.794(6)	6.3655(20)	9.646(3)	117.302(5)	0.5000		0.72054	0.991	0.004	0.000	0.002	0.002	0.000	0.001	0.47
																
(34)‡	Mg-wolfeite, Yukon, USA	13.010(4)	6.585(3)	9.754(2)	116.62(3)	0.5000		0.77400	0.100	0.825	0.075	0.000	0.000	0.000	0.000	0.05
(35)	Triplite, Canyon City, USA	13.1728(16)	6.4429(7)	9.9264(12)	118.927(6)	0.36536(5)		0.76073	0.321	0.144	0.518	0.014	0.002	0.000	0.000	0.88
(36)	Zwieselite, Olary Block, Australia	13.1770(3)	6.5020(1)	9.9523(2)	118.8378	0.40043(8)		0.77720	0.047	0.555	0.382	0.014	0.002	0.000	0.000	0.75
(37)	Triplite, Chanteloube, France	13.304(3)	6.508(2)	10.032(3)	119.478(5)	No sat. ref.		0.77957	0.007	0.426	0.526	0.038	0.002	0.000	0.000	0.84
(38)	Triplite, Mica Lode, USA	13.12036(30)	6.4575(15)	9.9511(22)	119.051(4)	0.3656(8)		0.76291	0.284	0.149	0.549	0.015	0.003	0.000	0.000	0.89
(39)	Zwieselite, Hagendorf, Germany	13.1957(18)	6.4889(9)	9.9764(8)	119.210(7)	†	(1*b*)	0.77912	0.015	0.591	0.376	0.016	0.003	0.000	0.000	0.83

Source of information on occurrence: (1) Vry Cartwright (1994 ▶); (2) Ouzegane *et al.* (2003 ▶); (3) and (4) Izbrodin *et al.* (2008 ▶); (5) Novk Povondra (1984 ▶); (6) Wight Chao (1995 ▶); (7) Chesnokov *et al.* (2008 ▶); (8) Jaffe *et al.* (1992 ▶); (9) Grew *et al.* (2006 ▶); (10) Simmat Rickers (2000 ▶); (11) Ginzburg *et al.* (1951 ▶); (12) Gonzlez del Tnago Peinado (1992 ▶); (13) Ginzburg *et al.* (1951 ▶); (14) Henriques (1956 ▶); (15) Fontan (1981 ▶); (16) Keller, Fransolet Fontan (1994 ▶), Keller, Fontan Fransolet (1994 ▶); (17) Corbella Melgarejo (1990 ▶); (18) Ren *et al.* (2003 ▶); (19) Wyss (1999 ▶); (20) Roda *et al.* (2004 ▶); (21) Kirchner (1982 ▶); (22) Braitsch (1960 ▶); (23) Sheridan *et al.* (1976 ▶); (24) Roy *et al.* (2003 ▶); (25) Grew *et al.* (2000 ▶); (26) and (27) Hegemann Steinmetz (1927 ▶); (28) Nijland *et al.* (1998 ▶); (29) Irouschek-Zumthor Armbruster (1985 ▶); (30) Hejny Armbruster (2002 ▶); (31) Leroux Ercit (1992 ▶); (32) Kelly Rye (1979 ▶); (33) Brunet *et al.* (1998 ▶); (34) Kolitsch (2003 ▶); (35) Heinrich (1951 ▶); (36) Lottermoser Lu (1997 ▶); (37) Otto (1935 ▶); (38) Heinrich (1951 ▶); (39) Keller, Fransolet Fontan (1994 ▶), Keller, Fransolet Fontan (1994 ▶).

Source of samples: (1) Julie Vry, X220; (2) J.-R. Kienast, In928; (3) and (4) Fersman Museum #62065; (5) Milan Novk; (6) Canadian Museum of Nature #83763; (7) B. V. Chesnokov #054-473; (8) National Museum of Natural History #170977; (9) E. S. Grew #10508; (10) Ralf Simmat, X-4; (11) P. M. Kartashov; (12) J. Gonzlez del Tnago from pegmatite #30; (13) Fersman Museum #50653; (14) Ecole des Mines de Paris #16926; (15) Ecole des Mines de Paris #41494; (16) P. Keller, TSAO-103; (17) J. C. Melgarejo; (18) Liudong Ren; (19) Nicolas Meisser, Muse Gologie Lausanne #8.BB.15; (20) F. Fontan; (21) E. Kirchner; (22) University of Gttingen; (23) National Museum of Natural History #160005; (24) E. S. Grew #11412; (25) E. S. Grew #12213; (26) E. Kirchner; (27) Naturhistorisches Museum Bern #A2606; (28) Ecole des Mines de Paris #38513; (29) University of Bern; (30) South Australia Museum #616351; (31) Marc Leroux SC-5-21F; (32) Staatssammlung Mnchen #27901; (33) C. Chopin 85DM73c; (34) U. Kolitsch; (35) American Museum of Natural History #21326; (36) American Museum of Natural History #91609; (37) Ecole des Mines de Paris #16925; (38) Harvard University Mineralogical Museum # 97893; (39) Ecole des Mines de Paris #36158.

† Crystals were mesured on an EnrafNonius (CAD4) diffractometer with a conventional point detector. The periodicity of the structures was determined using diagnostic ‘fingerprint’ reflections whose *hkl* indices correspond to the superstructures and not to the basic (1*b*) type.

‡ The cell parameters and chemical compositions of these samples are taken from the cited papers and recalculated in terms of our settings and formula units. Data on all the other samples were obtained in the present study.

**Table 3:** Correlation between + and motifs observed in HRTEM images of (5*b*), (7*b*) and (9*b*) wagnerites with U (up) and D (down) arc-like arrangements of F, OH of the wagnerite structures projected along *c*

Wagnerite	[+ ] sequence	[U D] sequence
(5*b*)	[+ + + ]	[U U D U D]
(7*b*)	[+ + + + ]	[U U D U D U D]
	[+ + + ]	[U D U D D U D]
(9*b*)	[+ + + + + ]	[U U D U D U D U D]

**Table 4:** Experimental details For all structures: *Z* = 8. Experiments were carried out at 293K with Mo*K* radiation using a Bruker CCD diffractometer. Absorption was corrected for by multi-scan methods, *SADABS* (Bruker, 2011 ▶).

Wagnerite from	Panasqueira, Portugal (3*b*)	Hlsjberg, Sweden (5*b*)	Khyakhta, Russia (orange) (7*b*)	Reynolds Range, Australia (9*b*)	Webing, Austria (2*b*)
Crystal data					
Chemical formula	Mg_0.8_Fe_0.5_Mn_0.7_(PO_4_)F_0.8_(OH)_0.2_	Mg_1.3_Fe_0.5_Mn_0.2_(PO_4_)F_0.7_(OH)_0.3_	Mg_1.7_Fe_0.25_Mn_0.05_(PO_4_)F_1.0_	Mg_1.94_Fe_0.06_F_0.98_(OH)_0.02_	Mg_2_(PO_4_)F
*M*_r_	196.6	183	168.43	163.5	162.6
Crystal system	Monoclinic	Monoclinic	Monoclinic	Monoclinic	Monoclinic
Space group	*C*2/*c*(00)*s*0	*C*2/*c*(00)*s*0	*C*2/*c*(00)*s*0	*C*2/*c*(00)*s*0	*C*2/*c*(00)*s*0
Wavevectors	**q** = 0.345990b*	**q** = 0.410660b*	**q** = 0.427560b*	**q** = 0.446520b*	**q** = 0.500000b*
*a*, *b*, *c* ()	13.0183(2), 6.4149(1), 9.8411(1)	12.8840(2), 6.3889(1), 9.7384(1)	12.7978(2), 6.3523(1), 9.6642(1)	12.7707(2), 6.3394(1), 9.6462(1)	12.7633(4), 6.3282(2), 9.6350(3)
()	118.562 (1)	117.799 (1)	117.567 (1)	117.5240 (5)	117.5985 (11)
*V* (^3^)	721.82(2)	709.10(2)	696.46(2)	692.55(2)	689.66(4)
(mm^1^)	4.99	3.47	1.8	1.18	1.07
Crystal size	0.160.160.06	0.20.150.15	0.60.160.1	0.250.250.1	0.460.260.26
					
Data collection					
No. of measured, independent and observed [*I* > 3(*I*)] reflections	9151, 2484, 2086	6943, 2439, 2216	17606, 7370, 3766	26453, 7409, 4855	12149, 3160, 2999
*R*_int_	0.011	0.010	0.016	0.010	0.009
(sin/)_max_ (^1^)	0.650	0.649	0.715	0.715	0.715
No. of satellite reflections					
First-order obs/all	1262/1653	1413/1625	1684/2106	1847/2106	1939/2102
Second-order obs/all		10%	344/2102	849/2122	
Third-order obs/all		10%	721/2108	1059/2119	
					
Refinement					
No. of reflections	2484	2439	7370	7409	3160
No. of parameters	167	228	504	503	227
No. of constraints	2	2	2	2	0
*R*_int_ obs/all	0.010/0.009	0.010/0.010	0.016/0.018	0.010/0.009	0.009/0.009
*R* obs/all	0.022/0.025	0.023/0.024	0.031/0.061	0.033/0.048	0.016/0.017
Main ref. *R* obs/all	0.019/0.019	0.022/0.022	0.024/0.025	0.023/0.023	0.018/0.018
First-order sat. *R* obs/all	0.032/0.046	0.025/0.030	0.027/0.038	0.020/0.025	0.014/0.016
Second-order sat. *R* obs/all		Not refined	0.128/0.391	0.197/0.305	
Third-order sat. *R* obs/all		Not refined	0.106/0.242	0.111/0.171	
*wR*_2_ obs/all	0.063/0.064	0.064/0.065	0.079/0.091	0.099/0.104	0.054/0.055
GOF obs/all	0.019/0.019	0.022/0.021	0.021/0.016	0.029/0.024	0.020/0.020

## Footnotes

1 Supporting information for this paper is available from the IUCr electronic archives (Reference: DK5018).
