# Supplementary material for: Superspace description of wagnerite-group minerals (Mg,Fe,Mn)2(PO4)(F,OH)
Source: Acta Crystallogr B Struct Sci Cryst Eng Mater. 2014 Mar 4;70(Pt 2):243–58. doi: 10.1107/S2052520613031247 (PMC3970752; doi:10.1107/S2052520613031247)
Supplement: Supplementary file 8 [file b-70-00243-sup8.pdf]

Table 5. Selected bond distances, including average (ave.) and extreme (min. and max.) caused by modulation in the structure of wagnerite from Panasqueira, Portugal.

Bonds	ave. (Å)	min. (Å)	max. (Å)	symmetry codes
t = 0 - 0.53				
M1-F1	2.100(6)	2.056(4)	2.180(7)	
M1-O1	2.1544(12)	2.1188(12)	2.1751(12)	
M1-O1	2.0847(10)	2.0791(11)	2.0936(10)	-x,-y+1,-z
M1-O3	2.1027(17)	2.0846(17)	2.1134(17)	
M1-O4	2.1432(10)	2.1382(10)	2.1464(10)	-x+1/2,y-1/2,-z+1/2
M1-F2	2.324(8)	2.295(12)	2.334(3)	-x,-y,-z<br>(only for t = 0 - 0.47)
M2-F1	1.985(6)	1.920(9)	2.022(3)	
M2-F1	2.169(5)	2.165(5)	2.177(5)	-x,y,-z+1/2
M2-O2	2.0727(12)	2.0696(12)	2.0794(12)	-x,y,-z+1/2
M2-O2	2.1012(10)	2.0845(10)	2.1378(10)	x-1/2,-y+1/2,z-1/2
M2-O3	2.0658(12)	2.0591(12)	2.0731(12)	-x,-y,-z
M2-O4	2.0320(17)	2.0227(17)	2.0533(17)	x-1/2,y-1/2,z
t=0.53-1				
M1-F2	2.034(11)	2.019(16)	2.045(5)	
M1-O1	2.1016(13)	2.0895(13)	2.1272(12)	
M1-O1	2.0886(11)	2.0807(10)	2.0937(10)	-x,-y+1,-z
M1-O3	2.0740(17)	2.0671(17)	2.0878(17)	
M1-O4	2.1431(10)	2.1384(10)	2.1454(10)	-x+1/2,y-1/2,-z+1/2
M2-F1	2.182(3)	2.169(4)	2.192(4)	-x,y,-z+1/2
M2-F2	2.047(12)	2.031(6)	2.086(18)	
M2-O2	2.0804(12)	2.0743(12)	2.0830(12)	-x,y,-z+1/2
M2-O2	2.1316(10)	2.0974(10)	2.1477(10)	x-1/2,-y+1/2,z-1/2
M2-O3	2.0680(12)	2.0596(12)	2.0731(12)	-x,-y,-z
M2-O4	2.0619(17)	2.0398(17)	2.0724(17)	x-1/2,y-1/2,z
t = 0-1				
P1-O1	1.5314(10)	1.5281(10)	1.5361(10)	
P1-O2	1.5390(13)	1.5344(13)	1.5438(14)	
P1-O3	1.5424(14)	1.5359(14)	1.5489(14)	x,-y+1,z+1/2
P1-O4	1.5325(16)	1.5282(16)	1.5364(15)	

*The bond calculation run for t from the interval <0.000,0.500> at 51 equidistant steps

Table 6. Selected bond distances, including average (ave.) and extreme (min. and max.) caused by modulation in the structure of wagnerite from from Hålsjöberg, Sweden.

Bonds	ave. (Å)	min. (Å)	max. (Å)	symmetry codes
t=0-0.5				
M1-F1	2.071(7)	2.052(5)	2.104(10)	
M1-F2	2.240(5)	2.191(2)	2.343(8)	-x,-y,-z
M1-O1	2.1664(12)	2.0993(12)	2.2050(12)	
M1-O1	2.0738(10)	2.0706(10)	2.0792(10)	-x,-y+1,-z
M1-O3	2.0971(16)	2.0709(16)	2.1122(16)	
M1-O4	2.1302(10)	2.1109(10)	2.1411(10)	-x+1/2,y-1/2,-z+1/2
M2-F1	2.188(6)	2.178(6)	2.198(6)	-x,y,-z+1/2
M2-O2	2.0566(11)	2.0474(11)	2.0726(11)	-x,y,-z+1/2
M2-O2	2.0683(10)	2.0402(10)	2.1343(10)	x-1/2,-y+1/2,z-1/2
M2-O3	2.0417(11)	2.0288(11)	2.0597(11)	-x,-y,-z
M2-O4	2.0261(16)	2.0119(16)	2.0600(16)	x-1/2,y-1/2,z
t=0.5-1				
M1-F2	1.978(7)	1.915(11)	2.017(3)	
M1-O1	2.0640(12)	2.0403(13)	2.1167(12)	
M1-O1	2.0798(10)	2.0753(10)	2.0811(10)	-x,-y+1,-z
M1-O3	2.0562(16)	2.0466(16)	2.0770(16)	
M1-O4	2.1203(10)	2.1105(10)	2.1336(10)	-x+1/2,y-1/2,-z+1/2
M2-F1	2.083(4)	2.021(2)	2.198(6)	-x,y,-z+1/2
M2-F2	2.032(8)	2.009(4)	2.080(12)	
M2-O2	2.0877(11)	2.0714(11)	2.0972(11)	-x,y,-z+1/2
M2-O2	2.1299(10)	2.0635(10)	2.1615(10)	x-1/2,-y+1/2,z-1/2
M2-O3	2.0534(11)	2.0313(11)	2.0634(11)	-x,-y,-z
M2-O4	2.0823(16)	2.0391(16)	2.1046(16)	x-1/2,y-1/2,z
t=0-1				
P1-O1	1.5335(10)	1.5284(10)	1.5420(10)	
P1-O2	1.5388(12)	1.5311(12)	1.5474(13)	
P1-O3	1.5415(13)	1.5333(13)	1.5495(13)	x,-y+1,z+1/2
P1-O4	1.5335(14)	1.5265(14)	1.5399(14)	

*The bond calculation run for t from the interval <0.000,0.500> at 51 equidistant steps

Table 7. Selected bond distances, including average (ave.) and extreme (min. and max.) caused by modulation in the structure of wagnerite from Kyakhta, Rusia.

Bonds	ave. (Å)	min.(Å)	max.(Å)	symmetry codes
t=0-0.5				
M1-F1	2.027(3)	2.001(3)	2.080(3)	
M1-F2	2.221(2)	2.1815(19)	2.334(2)	-x,-y,-z
M1-O1	2.155(3)	2.088(3)	2.200(3)	-x,-y+1,-z
M1-O1	2.063(2)	2.044(2)	2.083(2)	
M1-O3	2.080(3)	2.059(3)	2.089(3)	
M1-O4	2.095(2)	2.078(2)	2.108(2)	-x+1/2,y-1/2,-z+1/2
M2-F1	1.943(3)	1.858(3)	1.976(2)	
M2-O2	2.030(3)	2.015(3)	2.052(3)	-x,y,-z+1/2
M2-O2	2.053(2)	2.020(2)	2.116(2)	x-1/2,-y+1/2,z-1/2
M2-O3	2.023(3)	2.015(3)	2.039(3)	-x,-y,-z
M2-O4	2.012(3)	1.997(3)	2.049(3)	x-1/2,y-1/2,z
t=0.5 -1				
M1-F2	1.955(2)	1.879(3)	1.981(3)	
M1-O1	2.037(3)	2.012(3)	2.092(3)	
M1-O1	2.056(2)	2.043(2)	2.072(2)	-x,-y+1,-z
M1-O3	2.046(3)	2.033(3)	2.064(3)	
M1-O4	2.096(2)	2.082(2)	2.108(2)	-x+1/2,y-1/2,-z+1/2
M2-F1	1.860(3)	1.858(3)	1.862(3)	-x,y,-z+1/2
M2-F2	2.030(3)	2.010(3)	2.088(3)	
M2-O2	2.078(3)	2.048(3)	2.103(3)	-x,y,-z+1/2
M2-O2	2.118(2)	2.059(2)	2.170(2)	x-1/2,-y+1/2,z-1/2
M2-O3	2.046(3)	2.025(3)	2.057(3)	-x,-y,-z
M2-O4	2.078(3)	2.028(3)	2.104(3)	x-1/2,y-1/2,z
t=0-1				
P1-O1	1.533(2)	1.523(2)	1.544(2)	
P1-O2	1.540(3)	1.531(3)	1.548(3)	
P1-O3	1.539(3)	1.531(3)	1.549(3)	x,-y+1,z+1/2
P1-O4	1.535(3)	1.529(3)	1.546(3)	

*The bond calculation run for t from the interval <0.000,0.500> at 51 equidistant steps

Table 8. Selected bond distances, including average (ave.) and extreme (min. and max.) caused by modulation in the structure of wagnerite from Reynolds Range, Australia.

Bonds	ave. (Å)	min.(Å)	max.(Å)	symmetry codes
t = 0-0.5				
M1-F1	2.013(2)	1.984(2)	2.072(2)	
M1-F2	2.2152(19)	2.1758(16)	2.330(2)	-x,-y,-z
M1-O1	2.151(2)	2.082(2)	2.196(2)	
M1-O1	2.0610(19)	2.0429(19)	2.0820(19)	-x,-y+1,-z
M1-O3	2.077(3)	2.057(3)	2.086(3)	
M1-O4	2.0791(18)	2.0604(18)	2.0950(18)	-x+1/2,y-1/2,-z+1/2
M2-F1	1.938(2)	1.852(2)	1.9737(19)	
M2-O2	2.020(2)	2.000(2)	2.049(2)	-x,y,-z+1/2
M2-O2	2.0507(17)	2.0193(17)	2.1089(18)	x-1/2,-y+1/2,z-1/2
M2-O3	2.017(2)	2.009(2)	2.030(2)	-x,-y,-z
M2-O4	2.010(3)	1.995(3)	2.046(3)	x-1/2,y-1/2,z
t=0.5-1				
M1-F2	1.940(2)	1.860(2)	1.968(2)	
M1-O1	2.031(2)	2.005(2)	2.086(2)	
M1-O1	2.0484(19)	2.0327(19)	2.0619(19)	-x,-y+1,-z
M1-O3	2.045(3)	2.033(3)	2.059(3)	
M1-O4	2.0866(18)	2.0683(18)	2.0985(18)	-x+1/2,y-1/2,-z+1/2
M2-F1	2.0749(14)	2.0324(13)	2.1791(15)	-x,y,-z+1/2
M2-F2	2.037(2)	2.018(2)	2.103(3)	
M2-O2	2.077(2)	2.046(2)	2.105(2)	-x,y,-z+1/2
M2-O2	2.1113(17)	2.0529(17)	2.1657(17)	x-1/2,-y+1/2,z-1/2
M2-O3	2.044(2)	2.023(2)	2.057(2)	-x,-y,-z
M2-O4	2.075(3)	2.029(3)	2.099(3)	x-1/2,y-1/2,z
t=0-1				
P1-O1	1.5327(17)	1.5250(17)	1.5448(17)	
P1-O2	1.539(2)	1.531(2)	1.546(2)	
P1-O3	1.538(2)	1.528(2)	1.549(2)	x,-y+1,z+1/2
P1-O4	1.535(3)	1.530(3)	1.544(2)	

*The bond calculation run for t from the interval <0.000,0.500> at 51 equidistant steps

Bond	ave. (Å)	min. (Å)	max. (Å)	symmetry codes
t = 1/8, 5/8				
Mg1-F1	1.9939(8)	1.9939(8)	1.9939(8)	
Mg1-F2	1.9422(7)	1.9422(7)	1.9422(7)	
Mg1-F2	2.2411(3)	2.2411(3)	2.2411(3)	-x,-y,-z
Mg1-O1	2.0867(4)	2.0125(4)	2.1610(4)	
Mg1-O3	2.0590(5)	2.0392(5)	2.0788(5)	
Mg1-O1	2.0482(4)	2.0447(4)	2.0516(4)	-x,-y+1,-z
Mg1-O4	2.0745(3)	2.0569(3)	2.0920(3)	-x+1,y-1,-z+1/2
Mg2-F1	1.9536(7)	1.9536(7)	1.9536(7)	
Mg2-F1	2.0578(5)	2.0578(5)	2.0578(5)	-x,y,-z+1/2
Mg2-F2	2.0372(8)	2.0372(8)	2.0372(8)	
Mg2-O2	2.0421(4)	1.9945(4)	2.0897(4)	-x,y,-z+1/2
Mg2-O2	2.0777(4)	2.0774(4)	2.0781(4)	x-1,-y,z-1/2
Mg2-O3	2.0285(4)	2.0181(4)	2.0389(4)	-x,-y,-z
Mg2-O4	2.0437(5)	2.0033(5)	2.0841(5)	x-1,y-1,z
P1-O1	1.5344(3)	1.5263(3)	1.5425(3)	
P1-O2	1.5408(4)	1.5373(4)	1.5442(4)	
P1-O4	1.5381(4)	1.5339(4)	1.5422(4)	
P1-O31	1.5379(4)	1.5344(4)	1.5414(4)	x-1,-y+1,z+1/2

Table 9. Selected bond distances, including average (ave.) and extreme (min. and max.) caused by modulation in the structure of wagnerite from Webing, Austria.

t = 3/8, 9/8				
Mg1-F1	1.9920(4)	1.9920(4)	1.9920(4)	
Mg1-F2	1.9459(4)	1.9459(4)	1.9459(4)	
Mg1-F2	2.1988(5)	2.1988(5)	2.1988(5)	-x,-y,-z
Mg1-O1	2.0481(4)	2.0277(4)	2.0685(4)	-x,-y+1,-z
Mg1-O4	2.0776(4)	2.0763(4)	2.0789(4)	-x+1,y,-z+1/2
Mg1-O1	2.0813(4)	2.0340(4)	2.1286(4)	
Mg1-O3	2.0616(5)	2.0478(5)	2.0755(5)	
Mg2-F1	1.9371(4)	1.9371(4)	1.9371(4)	
Mg2-F1	2.0728(3)	2.0728(3)	2.0728(3)	-x+1,y,-z+1/2
Mg2-F2	2.0198(5)	2.0198(5)	2.0198(5)	
Mg2-O3	2.0263(4)	2.0046(4)	2.0479(4)	-x,-y,-z
Mg2-O4	2.0431(5)	2.0144(5)	2.0718(5)	
Mg2-O2	2.0424(4)	2.0229(4)	2.0619(4)	-x+1,y,-z+1/2
Mg2-O2	2.0813(4)	2.0227(3)	2.1398(4)	x-1,-y+1,z-1/2
P1-O3	1.5374(4)	1.5284(4)	1.5464(4)	x,-y+1,z+1/2
P1-O1	1.5304(3)	1.5275(3)	1.5332(3)	
P1-O2	1.5370(4)	1.5294(4)	1.5446(4)	
P1-O4	1.5324(4)	1.5293(4)	1.5356(4)	

*The bond calculation run for t from the interval <0.125,0.625> at 2 equidistant steps

Table 10. Selected bond distances in the structure of wagnerite from Webing, Austria (3D model).

Bonds	(Å)	symmetry codes
Mg1-O6	2.0131(5)	
Mg1-O12	2.0068(4)	
Mg1-O13	2.0281(4)	x+1,y,z+1
Mg1-O13	2.0186(4)	-x+1,-y,-z
Mg1-F1	1.9414(5)	
Mg2-O5	2.0703(5)	
Mg2-O11	2.0494(4)	
Mg2-O14	2.1433(4)	
Mg2-O14	2.0583(4)	
Mg2-F1	2.0696(4)	
Mg2-F2	2.0257(5)	
Mg3-O7	2.0010(5)	
Mg3-O10	2.0172(4)	
Mg3-O15	1.9960(4)	x+1/2,-y+1/2,z+1/2
Mg3-O16	2.0709(4)	x+1,y,z+2
Mg3-F4	1.9517(5)	x,y,z+1
Mg4-O8	2.0835(5)	
Mg4-O9	2.0368(4)	
Mg4-O15	2.0740(4)	x,y,z-1
Mg4-O16	2.0923(4)	x+1/2,-y+1/2,z+1/2
Mg4-F3	2.0308(5)	x,y,z-1
Mg4-F4	2.0606(4)	
Mg5-O1	2.0294(4)	
Mg5-O3	2.0339(4)	
Mg5-O6	2.0790(4)	-x+1,-y,-z
Mg5-O10	2.0501(5)	-x+1,-y,-z+1
Mg5-F2	1.9446(5)	-x+1,-y,-z
Mg6-O2	2.0698(4)	
Mg6-O4	2.1284(4)	
Mg6-O5	2.0813(4)	-x+1,-y,-z
Mg6-O9	2.0780(5)	-x+1,-y,-z-1
Mg6-F1	1.9910(5)	-x+1,-y,-z
Mg6-F3	2.1992(4)	-x+1,-y,-z
Mg7-O1	2.0124(4)	
Mg7-O3	2.0498(4)	
Mg7-O7	2.0914(4)	x-1/2,-y+1/2,z-1/2
Mg7-O12	2.0365(5)	x-1/2,-y+1/2,z-1/2
Mg7-F3	1.9443(5)	x-1/2,-y+1/2,z-1/2
Mg8-O2	2.1611(4)	
Mg8-O4	2.0429(4)	
Mg8-O8	2.0566(4)	x-1/2,-y+1/2,z-1/2
Mg8-O11	2.0765(5)	x-1/2,-y+1/2,z-1/2

Mg8-F2	2.2394(4)	x-1/2,-y+1/2,z-1/2
Mg8-F4	1.9936(5)	x-1/2,-y+1/2,z-1/2

Table 11. Selected bond distances in the structure of wagnerite from Webing, Austria (3D model).

Wagnerite from Webing - 3D model		
Bonds	(Å)	symmetry codes
P1-O1	1.5410(3)	
P1-O5	1.5302(5)	
P1-O9	1.5341(4)	-x+1,-y,-z
P1-O15	1.5359(4)	
P2-O2	1.5255(3)	
P2-O6	1.5397(4)	x-1,y,z-1
P2-O10	1.5410(4)	-x+1,-y,-z
P2-O16	1.5438(4)	
P3-O3	1.5357(3)	
P3-O7	1.5371(5)	x-1,y,z-1
P3-O11	1.5299(4)	x-1/2,-y+1/2,z-1/2
P3-O13	1.5320(4)	
P4-O4	1.5301(3)	
P4-O8	1.5307(5)	
P4-O12	1.5474(4)	x-1/2,-y+1/2,z-1/2
P4-O14	1.5461(4)	
